# Adaptive Genetic Algorithm for Optical Metasurfaces Design

**DOI:** 10.1038/s41598-018-29275-z

**Published:** 2018-07-23

**Authors:** Samad Jafar-Zanjani, Sandeep Inampudi, Hossein Mosallaei

**Affiliations:** Metamaterials Lab, Electrical and Computer Engineering Department, Northeastern University, Boston, Massachusetts, 02115 USA

## Abstract

As optical metasurfaces become progressively ubiquitous, the expectations from them are becoming increasingly complex. The limited number of structural parameters in the conventional metasurface building blocks, and existing phase engineering rules do not completely support the growth rate of metasurface applications. In this paper, we present digitized-binary elements, as alternative high-dimensional building blocks, to accommodate the needs of complex-tailorable-multifunctional applications. To design these complicated platforms, we demonstrate adaptive genetic algorithm (AGA), as a powerful evolutionary optimizer, capable of handling such demanding design expectations. We solve four complex problems of high current interest to the optics community, namely, a binary-pattern plasmonic reflectarray with high tolerance to fabrication imperfections and high reflection efficiency for beam-steering purposes, a dual-beam aperiodic leaky-wave antenna, which diffracts TE and TM excitation waveguides modes to arbitrarily chosen directions, a compact birefringent all-dielectric metasurface with finer pixel resolution compared to canonical nano-antennas, and a visible-transparent infrared emitting/absorbing metasurface that shows high promise for solar-cell cooling applications, to showcase the advantages of the combination of binary-pattern metasurfaces and the AGA technique. Each of these novel applications encounters computational and fabrication challenges under conventional design methods, and is chosen carefully to highlight one of the unique advantages of the AGA technique. Finally, we show that large surplus datasets produced as by-products of the evolutionary optimizers can be employed as ingredients of the new-age computational algorithms, such as, machine learning and deep leaning. In doing so, we open a new gateway of predicting the solution to a problem in the fastest possible way based on statistical analysis of the datasets rather than researching the whole solution space.

## Introduction

Despite their great potential conventional metasurfaces are currently suffering from a number of limitations that impede their widespread applicability^1–3^. Metasurfaces, metamaterilas with reduced dimensionality in one direction^4–7^, offer many advantages over bulky diffractive optical components, such as reduced size and cost. They therefore find applications in vast areas including: optical beam-steering^8,9^, lensing^5,10,11^, imaging^12,13^, and holography^14,15^. The de facto approach in designing metasurfaces has been to choose a canonical meta-element based on some considerations regarding the problem at hand, and then performing a systematic study to form a pre-compiled library of sub-wavelength nano-antennas that ideally cover the whole required range of optical responses, in terms of scattering phase and/or amplitude^16–18^. The library elements can then be employed to realize the intended phase and amplitude profiles for wavefront-forming and holography applications. Canonical nano-antennas are mainly made of plasmonic materials, high-index dielectrics, or a combination of both, and provide their sought optical behaviour by resonance or waveguiding mechanisms. These mechanisms usually give rise to limited spectral and angular performance of the nano-antennas and consequently the metasurfaces made of them. The remedies that have been proposed in the literature to overcome narrowband and angle-dependent performance of the conventional metasurfaces include: using multi-resonant nano-antennas^19,20^ and stacking a few metasurface layers^21–24^. Although these techniques show a lot of promise, they suffer from some disadvantages in turn and do not seem to be the ultimate solutions. For instance, the layers in stacked metasrufaces should be placed relatively far from each other to avoid coupling between them, leading to a considerable increase in the size of the device.

As the optical metasurfaces become more pervasive, the expected design goals imposed on them are becoming more complex. Examples of such complex design goals are discussed elaborately in this paper. In order to overcome the above-mentioned limitations and accommodate the current complex design goals imposed on the optical metasurfaces, the natural solution is the extension of the design domain to incorporate many more parameters and offer more degrees of freedom. Instead of canonical elements, such as rectangular patches or circular disks, more complex geometries are therefore starting to be investigated. As an example, binary-pattern (or pixelated) metasurfaces^1,25–31^ are drawing a lot of attention, thanks to the advancements in the nanofabrication technology, and also available computational resources that are required to design them. Due to the great advantages that binary metasurfaces offer, we use them as building metasurface elements in this paper.

At the same time, these complex geometries, including the binary metasurfaces to be investigated in this article, do not lend themselves well to analysis and make the design process very complicated^2,25,32^, as, they incorporate tens or even hundreds of parameters and the conventional systematic study (also called parametric study) is not feasible for such structures. Moreover, having an intuitive image of the solution domain is essential for every successful design procedure. With high-dimensional parameter domains, however, such valuable image becomes very hard to achieve.

In order to deal with large parameter spaces, designers usually take advantage of optimization techniques. There are two main categorises of optimization techniques: local and global optimizers^33^. Local optimizers are tightly bound to the solution domain and take advantage of initial guesses. However, due to this close bound, they impose some limitations, such as continuity, to the solution domain and the objective function. There is also a high chance for local optimizers to become stuck in a local extremum close to the initial guess, instead of the optimal global solution. Global optimizers, on the other hand, are mostly independent of the solution domain and therefore do not impose the mentioned constraints on the optimization problems. Problems of optimizing metasurfaces with complex geometries, such as binary patterns in this paper, involve discrete solution domains, and discontinuous or non-differentiable objective functions. Among global optimization techniques genetic algorithms (GAs) are the most suitable to handle such problems.

Genetic algorithms^33–35^ are stochastic search optimizers that are based on the concepts of evolution and natural selection. GAs are inherently parallel algorithms, which makes them able to take advantage of today’s parallel supercomputers to expedite the optimization task by a factor close to the number of parallel workers. GAs perform very well in handling multi-dimensional function domains, problems with discrete solution domains, and non-differentiable objective functions^33^. More details regarding the conventional GAs are given in section S-1 of the Supplementary Information.

Due to the above-mentioned advantages of GAs, researchers have recently begun to employ them to overcome the limitations of optical metasurfaces and unleash their enormous potential to replace, or even improve, the performance of refractive optical components^2,25,32^. Nevertheless, the investigations have mostly been limited to single-objective optimization problems and relatively small parameter domains. As it was previously mentioned, the design goals imposed on the metasurfaces are becoming progressively more demanding, which directly translates into multi-objective optimization problems.

In most of the multi-objective optimization problems, some of the design objectives are of higher priority compared to the others. Common procedure to take this disparity into consideration is assigning higher weights in the objective function to design objectives of higher priority^36^. However, based on our experience in optimizing the optical metasurfaces by GAs, using constant weights in a single optimization will not usually yield acceptable results. The issue is that giving even a small weight to the less significant objectives might cause GAs to become stray and make their convergence very slow. In this paper, we employ an alternative procedure called adaptive GA (AGA) to get over this limitation and obtain a near-optimal solution, in a reasonable optimization time. AGA has already been successfully utilized in a variety of disciplines, including but not limited to, power distribution systems^37^, photovoltaic cell design^38^, power inverter design^39^, energy efficiency optimization of communication networks^40^, path planning of robot systems^41^, logic circuits design^42^, printed circuit board manufacturing^43^, ultrasonic motor design^44^, inventory routing^45^, flexible manufacturing systems^46^, determination of crystal structure^47^, and also electromagnetics^36^, to tackle multi-objective optimization problems. More details about AGA is given in the following section.

In order to showcase the unique capabilities of the AGA technique to handle the challenges in designing optical metasurfaces, we successfully solve four novel but complex problems, which are currently of high interest in the optics community. First, we optimize a binary-pattern plasmonic metasurface to steer the incident beam to an arbitrary direction in space. While this geometry offers advantages, such as lower sensitivity to fabrication imperfections and more relaxed feature sizes, large number of parameters–which is the characteristic of all problems solved in this paper–makes its design very challenging. High reflection efficiencies are also difficult to achieve, due to the absorption loss of the plasmonic materials employed in this structure. To handle these challenges, we employ the AGA technique to first optimize the metasurface elements to provide specific phase retardations from 0 to 2*π*, and then update the fitness function to include a weight for the reflection amplitude. Next, we design a dual-beam leaky-wave antenna (LWA), which radiates transverse-electric (TE) and transverse-magnetic (TM) excitation waveguide modes to arbitrary directions. For this application, we optimize the whole binary array, in contrast to the previous case, where we only optimize the constitutive building blocks. The consequence is a considerable increase in the number of optimization parameters. GAs show a lot of promise in handling such high-dimensional function domains and can be considered to optimize very large devices, such as meta-lenses, at once if the required computational resources are available. Then, we optimize the binary asymmetrical topology of the constitutional nano-antennas of an all dielectric metasurface to deflect the perpendicular components of the incident light to arbitrary directions. Our goal in this application is to reduce the lateral dimensions of the metasurface inclusions, compared to the canonical elements. Finally, we design a visible-transparent Infrared (IR) emitting/absorbing metasurface by the AGA method. The proposed structure offers a great potential in areas such as solar-cell cooling. We employ a sophisticated combination of materials, including indium tin oxide (ITO) and fused silica, that show large absorption in the IR regime, and act as low-loss dielectrics in the visible spectrum. Furthermore, we optimize most of the geometric parameters of the array unit-cell to achieve broadband angle-independent absorption in IR and maximum transparency in visible.

Towards the end of this paper we present a unique way, utilizing GAs, to produce large datasets suitable to transfer to new-age computational algorithms, such as, machine learning and deep learning^48^. We present that, by analyzing the datasets using stochastic analysis, many inferences on the solution space could be derived without any extra computational costs. Such datasets will pave the way for new paradigm of predicting, instead of computing the required parameter set for a targeted solution.

## Adaptive Genetic Algorithm

**Figure 1:**
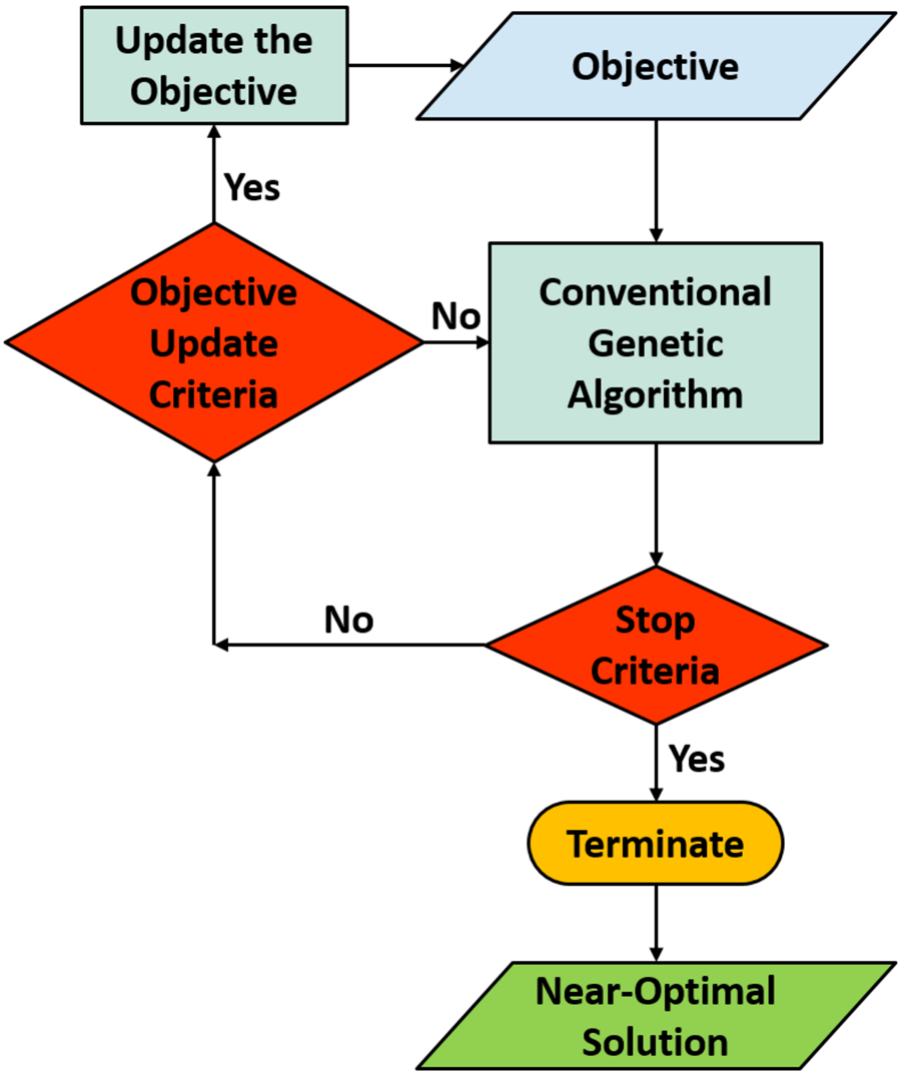
Flowchart for Adaptive Genetic Algorithm (AGA).

In this section we provide a brief introduction to the adaptive genetic algorithm (AGA) technique, which is employed to optimize the structures presented in this paper. In the simplest form an optimization problem to be solved with a GA can be expressed as

1 $$\mathop{{\rm{\max }}}\limits_{{\bf{p}}\in {\mathbb{R}}} {\mathcal F} ({\bf{p}})$$

where ${\mathcal F} ({\bf{p}})$ is called the objective or fitness function, and **P** = (*p*_1_, *p*_2_, ..., *p*_*n*_) is a vector representing the parameter space. It is worth mentioning that, although we employ GA for maximization purposes in this paper, GAs can also be successfully used in minimization problems^33^. Also, sometimes there are constraints that must be considered while maximizing the fitness function (e.g. constraints imposed by the fabrication process^25^), which we have ignored here for the sake of simplicity. For a multi-objective optimization problem the fitness function can be written in the most general form as

2 $$ {\mathcal F} ({\bf{p}})={W}_{1}\times {f}_{1}({\bf{p}})+{W}_{2}\times {f}_{2}({\bf{p}})+\mathrm{...}+{W}_{n}\times {f}_{n}({\bf{p}})$$

where ${f^{\prime} }_{i}s$ are multiple objective sub-functions and ${W^{\prime} }_{i}s$ are the corresponding weights. In a conventional multi-objective GA optimization, the weights corresponding to different objective functions in eq. 2 are constant throughout the optimization. The objective functions with higher priorities are given higher costs, compared to the less significant objectives^36^. This procedure, however, might cause GAs to become stray and deviated from the acceptable solutions in a reasonable optimization time. To overcome this issue, we use a technique called adaptive GA (AGA) in this paper. A flowchart of AGA is given in Fig. 1. We start by considering non-zero costs only for high-priority objectives (for instance, retardation phase with high accuracy). We also consider an *objective update criteria* for updating the objective function. The conventional GA is used to initialize a population and evolve it based on the initial objective function (see section S-1 of the Supplementary Information). At the end of each generation replacement iteration of the GA, if the overall stop criteria is met, the optimization will be terminated. Otherwise, the objective update criteria is checked and the objective function is updated if necessary. This technique allows the GA to first converge to a generation of individuals with satisfactory high-priority sub-objectives, and then try to improve the low-priority sub-objectives. This process will continue until the GA stop criteria is met.

## Results and Discussion

In this section we present four novel applications that are successfully solved with the AGA technique. Each of these problems is chosen carefully to represent the complexity of binary-pattern metasurface design and to highlight the advantages of the AGA technique. More specifically, all of them include high-dimensional parameter domains, and therefore, systematic study or trial-and-error are not feasible to design them.

### Binary-Pattern Reflectarray Metasurface for Beam-Steering

Reflectarray metasurfaces are commonly used in telecommunications to direct an incoming light beam to a desired direction. The common nanoscatterers utilized in designing plasmonic reflectarrays are elements with canonical shapes such as rectangular patches^17^. Around the resonance frequency, a typical rectangular plasmonic patch antenna sweeps a phase range of 220° within 20 nm change of its side length. The uncertainty of phase in a fabrication tolerance of, say, 5 nm is therefore around 55°. On the other hand, due to the absorption loss, reflection amplitudes for a considerable range of phases are very small. In this section we demonstrate an optimized binary-pattern metasurface designed by the AGA technique that overcomes the above shortcomings. The optimized metasurface shows high reflection efficiency and is robust in terms of fabrication imperfections.

Optimization of binary metasurfaces in the microwave spectrum by GAs has attracted a lot of attention in recent years^28,49^, because of the advantages that these geometries can offer (see Introduction). Ref.^49^, for instance, has employed a GA to minimize maximum field enhancement factor (MFEF) of a microwave reflectarray for high-power operating conditions. Here, we present optimized binary metasurfaces for telecommunication wavelengths in the near-IR regime, around 1.55 μm.

**Figure 2:**
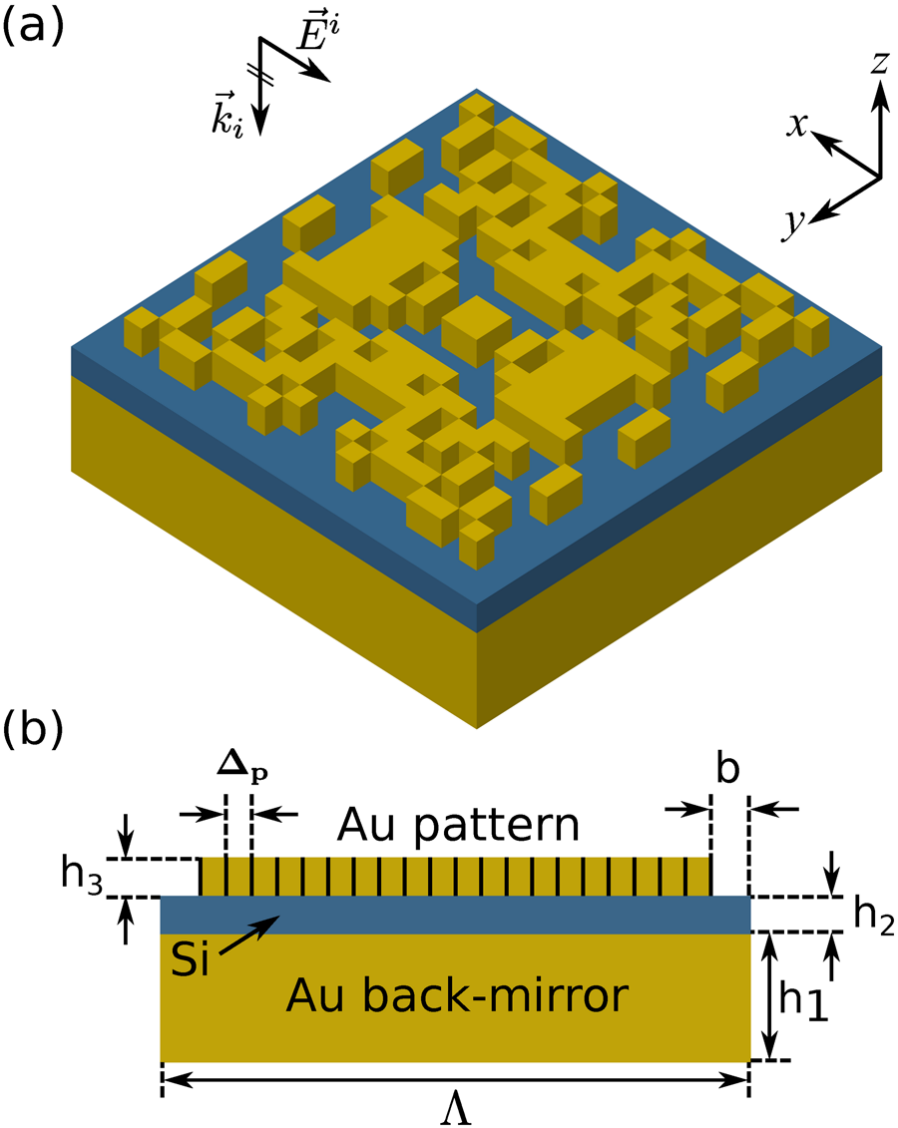
Unit-cell of the optimized plasmonic metasurface comprising a gold (Au) back-mirror, a silicon (Si) spacer layer and a binary gold pattern with 4-fold symmetry. One quarter of the binary pattern with 10 × 10 pixels is optimized. Operation frequency is 120 THz. (**a**) Perspective, and (**b**) side views of the unit-cell. Parameters: h_1_ = 100 nm, h_2_ = 30 nm, h_3_ = 30 nm, Δ_p_ = 25 nm, b = 25 nm, Λ = 550 nm.

A representative schematic of the binary unit-cell under consideration is depicted in Fig. 2. Periodicity and thickness of the layers have been considered as constant values, specified in the figure. The structure is illuminated by normal incidence of a plane wave (polarization of the incident wave is immaterial here, due to the 4-fold symmetry of the unit-cell). We optimize one quarter of the binary pattern with 10 × 10 pixels. Compared to the Metal-Insulator-Metal (MIM) patch widely employed for this application^17,18^, the binary nano-antenna of Fig. 2 has a resolution of 25 nm and is much more resilient to fabrication imperfections.

The fitness function that we use for this optimization is as follows:

3 $$ {\mathcal F} ({\bf{p}})={W}_{\phi }\times \exp [-6.0{(\phi ({\bf{p}})-{\phi }_{{\rm{target}}})}^{2}]+{W}_{a}\times \exp [-4.0{(1.0-A({\bf{p}}))}^{2}]$$

which is an example of the general fitness function of eq. 2, with two objective sub-functions. In ep.3, *φ*(**p**) and *A*(**p**) are phase and amplitude of the reflection coefficient, respectively, and *φ*_target_ is the targeted phase-delay. For this application, reflection-phase accuracy is of extreme importance. We therefore use a Gaussian function with a sharp peak as the phase objective. A smoother Gaussian function is chosen as the amplitude objective, as, perfect reflection amplitude close to 1.0 cannot be expected for some of the phase-delays in this application. More details regarding the choice of the fitness function are given in section 5.1.1 of the Supplementary Information.

The second term in eq. 3 is the amplitude objective sub-function. Although in this application reflection amplitude has lower priority, compared to phase-delay, it should still be considered as a sub-objective in the fitness function to ensure the highest reflection efficiency for the metasurface. One possible option is to consider a small value for *W*_*a*_ and perform a single optimization. However, this small value for *W*_*a*_ still might confuse the GA and result in optimized unit-cells which are not acceptable in terms of reflection phase. We therefore use AGA as a workaround to obtain both accurate reflection phase and high amplitude in a reasonable optimization time. More specifically, we first set *W*_*φ*_ = 1.0 and *W*_*a*_ = 0.0 and optimize the unit-cell just for retardation phase. After acceptable tolerance for phase has been achieved, we consider a small weight for amplitude (*W*_*φ*_ = 0.7 and *W*_*a*_ = 0.3) and continue optimization to ensure highest possible reflection efficiency. It is worth mentioning that, the specific value for *W*_*a*_ (relative to *W*_*φ*_) has been chosen empirically here, to yield GA convergence to solutions that are satisfactory in terms of both reflection phase and amplitude. Some general rules can be suggested, however, in this regard. The relative value of *W*_*a*_ should not be chosen too large to compensate for the lack of fitness in terms of the reflection phase accuracy, and deviate the GA population to contain high-ranking individuals which do not possess favorable reflection phases. It should not be chosen too small either to the extent that it may not have any tangible effect on GA convergence. Based on the physics of the problem at hand and relative priority of the sub-objectives, there might even be a range of values for the chosen relative weight of the second (and subsequent) sub-objective(s) that lead to satisfactory GA convergence.

**Figure 3:**
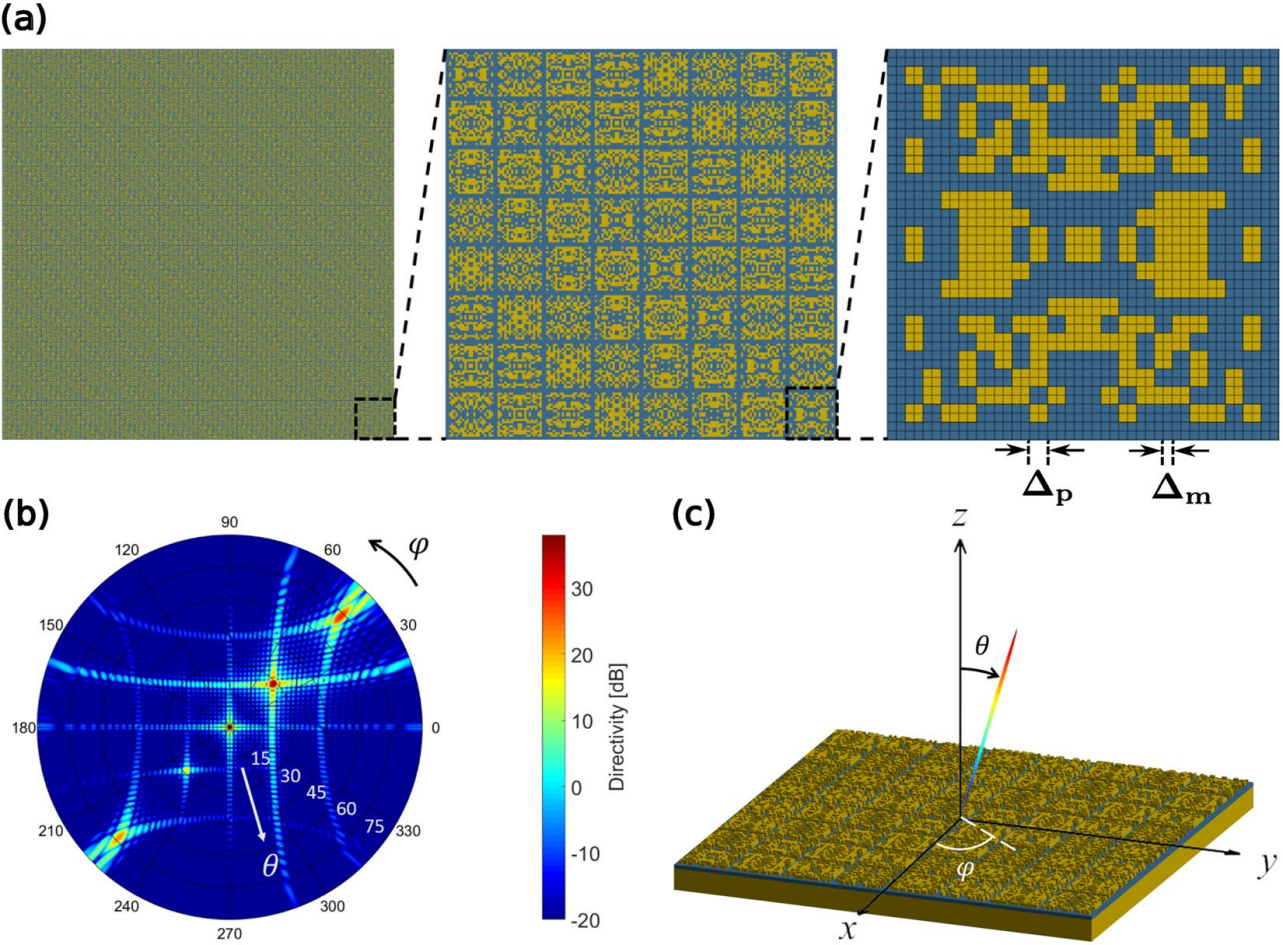
Performance of the optimized binary metasurface. (**a**) The image on the left shows the binary pattern of the designed metasurface to reflect a normally incident beam to (*θ*_0_, *φ*_0_) = (30°, 45°). The image in the middle shows the 8 × 8 super-cell of the metasurface and the right image shows the lower right unit-cell. Δ_m_ = 12.5 nm is the FDTD mesh size, and Δ_p_ = 2×Δ_m_ = 25 nm is the Au pixel size. (**b**) Two-dimensional reflection pattern of the designed metasurface, showing a main-lobe very close to the targeted direction at (*θ*, *φ*) = (28.6°, 45°). (**c**) A Three-dimensional linear reflection pattern of the designed reflectarray.

Following the procedure outlined above, we have designed a beam-steering metasurface to reflect a normally incident beam of light to the arbitrary direction of (*θ*_0_, *φ*_0_) = (30°, 45°). The required phase-delay distribution, over the aperture of the metasurface, is periodic in both lateral directions^17^. Accordingly, as it can be seen in the left panel of Fig. 3(a), the designed metasurface is a periodic arrangement of identical blocks in a 2-D square lattice. Each block is called a *super-cell* and we have considered the metasurface to be consisted of a 10 × 10 array of the super-cells. Each super-cell is in turn a 8 × 8 arrangement of smaller blocks, called unit-cells (see middle panel of Fig. 3(a)). There are 8 distinct unit-cells in a super-cell, and each unit-cell is a 4-fold symmetric pattern of Au pixels, designed by the AGA technique to introduce a specific phase-delay to the incident light, covering the whole 2 *π* range of possible phase-delays. Unit-cells are also optimized to have the maximum possible reflection amplitudes. The right panel of Fig. 3(a) shows one of the optimized unit-cells. More detail about designing the beam-steering metasurface is given in section S-2 of the Supplementary Information. Optimization parameters and statistics, along with the convergence plots for this application can also be found in sections S-5.1.2 and S-5.1.3 of the Supplementary Information, respectively. The designed metasurface is then characterized by a full-wave FDTD solver (see Methods) and the far-field reflection pattern is calculated by means of near-field to far-field transformation. The two-dimensional reflection pattern of the reflectarray is shown in Fig. 3(b). It can be seen in the figure that the designed array has a main-lobe very close to the targeted direction at (*θ*, *φ*) = (28.6°, 45°). Although a few side-lobes are present in the reflection pattern, they have much smaller levels, compared to the main-lobe. Moreover, as it has been shown in Fig. S-4 of the Supplementary Information, the metasurface of Fig. 3 possesses the 3 dB beamwidth of 2°. The linear three-dimensional reflection pattern of the metasurface is depicted in Fig. 3(c). It should be mentioned that, the small feature size of the Au pattern in the presented design (25 nm), and also the overall side-length of the optimized reflectarray, make the fabrication of the device, with the currently available technologies, such as electron-beam lithography, challenging. The high-resolution pattern is considered here to achieve accurate reflection phase for the optimized unit-cells, and showcase the capabilities of the AGA technique in optimizing the binary metasurfaces with a large number of structural parameters. Both of these parameters can be relaxed, however, in a new design, at the cost of some performance deterioration, in terms of reflection-phase accuracy of the optimized unit-cells and directivity of the reflectarray, respectively.

### Tailored Dual-Beam Aperiodic Optical Leaky-Wave Antenna

Conventional optical LWAs are periodic patterns inscribed on top of a planar slab waveguide to diffract the waveguide modes into a desired direction^50–52^. For a conventional LWA, the required periodicity to radiate an excitation waveguide mode to a specific direction (*θ*_0_) can be calculated by Λ = *λ*_0_/(*β*/*k*_0_ − *sinθ*_0_), where *β* is the phase constant of the excitation mode and Λ is the periodicity of the grating^50^. The phase constant, *β*, depends on the polarization of the excitation mode. Therefore, if the LWA is designed to radiate one of the excitation modes to a specific direction, a second excitation mode will automatically be radiated to another specific direction, defined by the same equation. In other words, the designer do not have the freedom to choose two arbitrary directions of radiation for two simultaneously present excitation modes with conventional periodic LWA’s (see section S-3.1 of the Supplementary Information for more details). We optimize an aperiodic grating in this section to overcome this limitation.

Unlike the previous application where we optimize the unit-cells of the metasurface array with specific retardation phases and highest possible reflection amplitudes, in this section, we present the results of optimizing the whole array of an LWA, intended to radiate transverse-electric (TE) and transverse-magnetic (TM) excitation waveguide modes to two arbitrarily desired directions. This application is chosen to showcase the capability of the AGA technique to optimize large aperiodic arrays if the required computational resources are available. Although we optimize a one-dimensional (1-D) grating here, the idea can be extended to computationally intensive 2-D and 3-D cases.

**Figure 4:**
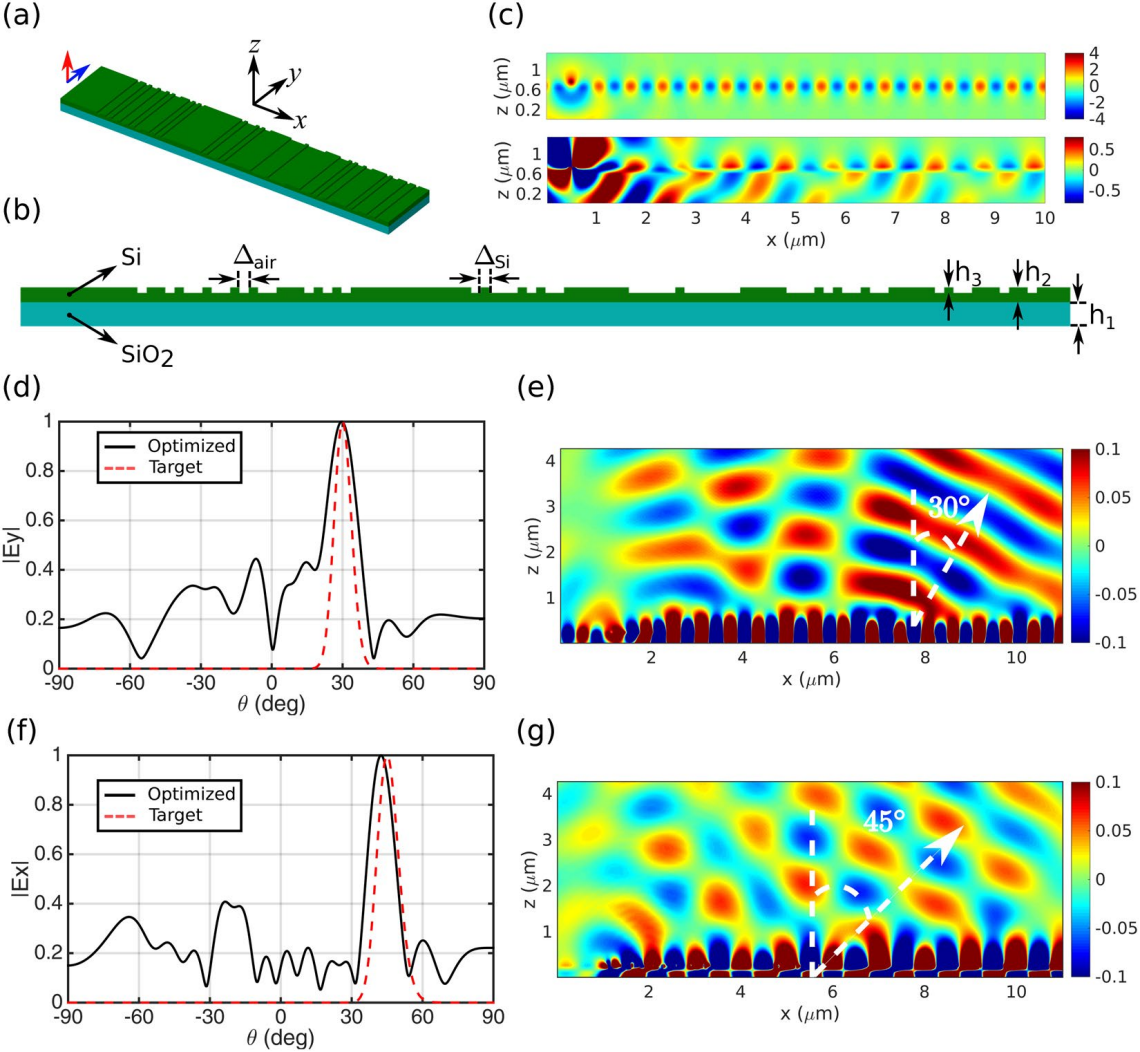
Geometry and performance of the optimized dual-beam LWA. (**a**) Perspective, and (**b**) top views of the optimized dual-beam LWA designed to radiated the TE excitation waveguide mode to *θ* = 30° and the TM mode to *θ* = 45°. Parameters: Δ_air_ = Δ_Si_ = 100 nm, h_1_ = ∞ (half-space), h_2_ = 160 nm, h_3_ = 60 nm. Operation frequency is 193.5 THz (*λ*_0_ = 1.55 μm). (**c)** The upper image shows the TE, and the lower image shows the TM excitation modes, respectively. (**d**) Far-field radiation pattern of the *y*-component of the electric field. (**e**) Near-field plot, showing the *y*-component of the electric field. (**f**) Far-field radiation pattern of the *x*-component of the electric field. (**g**) Near-field plot, showing the *x*-component of the electric field.

Figure 4(a) shows a schematic of the optimized LWA, consisting of a patterned silicon layer on top of a silica substrate. The antenna is fed by a combination of TE and TM waveguide modes. Excitation of waveguide modes with very high efficiency on silicon-on-insulator waveguides has been successfully demonstrated in the literature. As an example, Zhe *et al*.^53^ have recently proposed an optical coupler to efficiently couple light from a single-mode optical fiber to a silicon-on-insulator waveguide. Here, however, we use line sources instead to excite the modes, as a common practice in characterization of optical antennas. The upper image in Fig. 4(b) shows the TE waveguide mode generated by a *y*-polarized line-source, and the lower image shows the TM polarized waveguide mode, generated by a *z*-polarized line-source.

The fitness function that we use for this problem has the following form

4 $$ {\mathcal F} ({\bf{p}})={W}_{y}\times {f}_{y}({\bf{p}})+{W}_{x}\times {f}_{x}({\bf{p}})$$

where **p** is a vector representing the parameter space. Since the *y*-component of the electric field is generated only by the TE excitation mode and the *x*-component by the TM mode, we have considered two terms in the fitness function corresponding to *E*_*y*_ and *E*_*x*_ in the far-field region. We have considered the weights to be equal in this application (*W*_*y*_ = *W*_*x*_ = 0.5). Also, *f*_*y*_ and *f*_*x*_ sub-functions have the same form and, for instance, *f*_*y*_ can be written as

5 $${f}_{y}({\bf{p}})=(|{E}_{y}|({\bf{p}})\ast {|{E}_{y}|}^{{\rm{target}}})(\theta =\mathrm{0)}$$

where |*E*_*y*_| is radiation pattern of the *y*-component of the electric field for a specific set of parameters, **p**, and |*E*_*y*_|^target^ is the targeted radiation pattern with the main-lobe at the targeted radiation direction. Both |*E*_*y*_| and |*E*_*y*_|^target^ are functions of the elevation angle, *θ*. *f*_*y*_(**p**) is the zero-lag cross-correlation of |*E*_*y*_| and |*E*_*y*_|^target^, and the cross-correlation operator is normalized to return 1.0 if both function are the same and values less than 1.0 if they are different. More details regarding the utilized fitness function are presented in section 5.2.1 of the Supplementary Information.

Performance of the optimized LWA is also presented in Fig. 4. The antenna is optimized to difract the TE mode to *θ* = 30° and TM mode to *θ* = 45°. Figure 4(c) shows the radiation pattern of the *y*-component of the electric field, along with the targeted radiation pattern with maximum radiation at *θ* = 30°. Also, near-field plot of the *y*-component of the electric field is shown in Fig. 4(d). Similar plots for the *z*-component of the electric field are depicted in Fig 4(e) and (f) with targeted radiation direction at *θ* = 45°. For both components of the electric field the desired direction of radiation is achieved. As another example, the LWA is optimized to radiate the TE and TM modes to +45° and −45°, respectively, and the resutls are presented in section S-3.2 of the Supplementary Information. The optimization parameters, statistics, and the GA convergence plot are also reported in sections S-5.2.2 and S-5.2.3 of the Supplementary Information.

### Compact Birefringent All-Dielectric Metasurface Unit-Cells

A birefringent metasurface manipulates each polarization component of the incident light independently. Plasmonic rectangular patches in a square lattice^17^ and dielectric elliptical pillars in hexagonal lattices^54^ has been demonstrated in the literature to achieve such polarization based control on the propagation direction. While plasmonic metasurfaces utilize surface wave resonances of plasmonic elements, their all-dielectric counterparts employ waveguide modes of high-index dielectric materials to generate large ranges of phase-delay. Since the effective wavelength of the waveguide modes of canonical shapes is higher than the surface plasmon resonance modes, the required minimum building block lateral size in dielectric metasurfaces is always of the order of wavelength and cannot be reduced further. Consequently the resultant metasurfaces lose resolution in the bending angles^55^. One can verify that the minimum unit-cell dimension of a dielectric rectangular patch metasurface for complete birefringent control is around *λ*_0_/1.4 (with silicon spacer and grating height both equal to 100 nm, at *λ*_0_ = 900 nm). In this section, we demonstrate a reduction in the building block size to the order of *λ*_0_/2, by utilizing a binary dielectric pattern.

**Figure 5:**
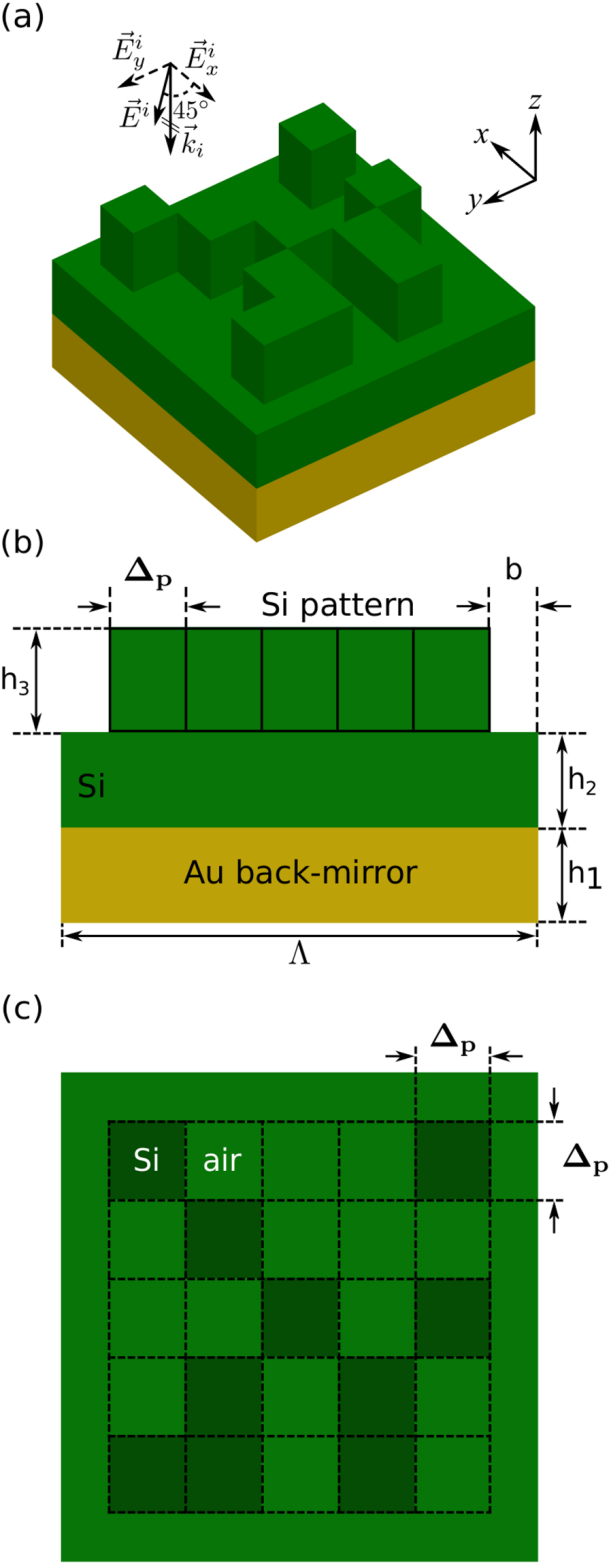
Unit-cell of the birefringent all-dielectric metasurface, consisting of a 5 × 5 asymmetrical pattern of silicon bricks on top of a silicon spacer layer, grounded by a gold back-mirror. (**a**) Perspective, (**b**) side, and (**c**) top views of the unit-cell. Parameters: h_1_ = 100 nm, h_2_ = 100 nm, h_3_ = 110 nm, Δ_p_ = 83 nm, b = 42 nm, Λ = 499 nm. Operation frequency is 327.87 THz (*λ*_0_ = 915 nm).

A schematic of the optimized unit-cell is depicted in Fig. 5. As it can be observed in Fig. 5 a normally incident plane wave with equal *x-* and *y-*components illuminates the structure. The goal in this problem is to design a metasurface that shows different functionalities for each perpendicular component of the incident filed. To achieve this goal, the unit-cell should introduce specific retardation phases to each electric field component. The fitness function is therefore defined as

6 $$ {\mathcal F} ({\bf{p}})={W}_{x}\times {f}_{x}({\bf{p}})+{W}_{y}\times {f}_{y}({\bf{p}})$$

where the first term is the objective sub-function corresponding to the *x*-component of the incident light and the second term corresponds to the *y*-component. Also, *f*_*x*_ and *f*_*y*_ have the same form, for instance,

7 $${f}_{y}({\bf{p}})=\exp [-\,6.0{({\phi }_{y}({\bf{p}})-{\phi }_{y}^{{\rm{target}}})}^{2}]$$

where *φ*_*y*_(**p**) is the retardation phase introduced to the *y*-component of the incident filed, corresponding to the parameter set **p**, and ${\phi }_{y}^{{\rm{target}}}$ is the targeted retardation phase for the *y*-component.

**Figure 6:**
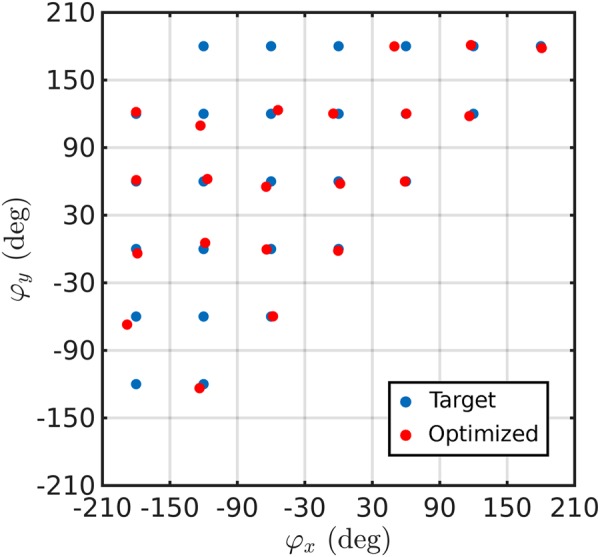
Optimized phase combinations for *x*- and *y*-components of the reflected electric field. Target phase combinations are also plotted (blue dots) for reference.

For each component of the electric field, we divide the whole retardation phase range from −180° to 180° to 6 intervals each of them with a 60° span, and consider the center of each interval as the optimization target. This way, we will have 36 combinations of phases for *x-* and *y-*components. However, based on the rotational symmetry of the polarizations and phase-wrapping, the 36 phase targets can be reduced to 26 (see Fig. 5). We have optimized the unit-cell to achieve these 26 phase combinations and plotted the results in Fig. 6 (red dots). Target phase combinations are also provided in the same figure for reference (blue dots). It can be seen in the figure that we have been able to obtain optimal solutions for most of the phase combinations, which is promising for many applications. Further details regarding the optimization parameters and statistics, along with the GA convergence plots can be found in section S-5.3.2 and S-5.3.3 of the Supplementary Information, respectively.

### Visible-Transparent Infrared-Emitting/Absorbing Metasurface

**Figure 7:**
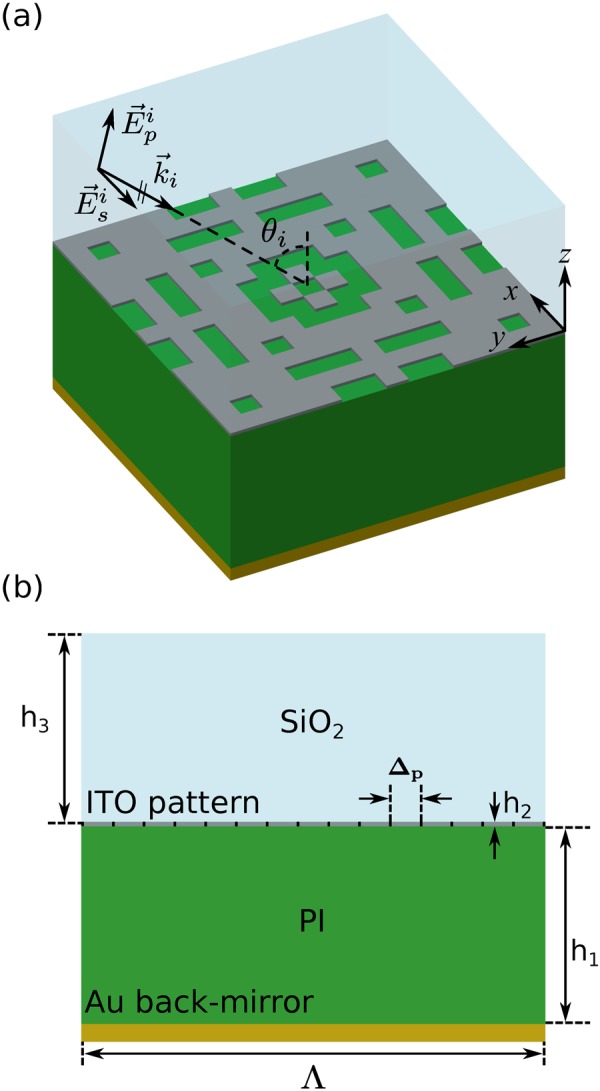
Unit-cell of the optimized visible-transparent infrared-emitting/absorbing metasurface, consisting of a gold back-mirror, a polyimide substrate, a patterned ITO layer with 8-fold symmetry, and a silica superstrate. The thickness of the ITO layer is considered to be 50 nm. Other dimensions of the unit-cell, including the periodicity, are optimized. Optimized parameters: h_1_ = 2.2 μm, h_3_ = 2.15 μm, Λ = 5.16 μm (Δ_p_ = Λ/15 = 344 nm).

In order to demonstrate the power of the AGA technique and its combination with FDTD, we handled a peculiar problem in this section that requires a unique binary pattern optimization for different functionalities at two separate frequency bands. We present an optimized pattern with large bandwidth of high absorptivity at IR frequencies and simultaneously high visible transparency. Such a metasurface layer can find potential applications in solar cell applications^56–61^ to filter out the unwanted thermal radiation that increases the temperature of the cell, without compromising the intensity of the visible frequency light. The idea is to add a layer to the conventional photovoltaic (PV) cell stack^62,63^, which absorbs the undesired thermal radiation, but, is transparent to visible light, and therefore does not interfere with the functionality of the PV cell. In that sense, the term “transparency” is used referring to the layer added to the conventional PV cell stack (ITO + SiO _2_ layer in Fig. 7) for cooling purposes. The optimization process also presents utilization of complex material models, intended to operate at multiple wavelength bands. It should be mentioned that, based on the Krickhoff’s law, at thermal equilibrium, the absorptivity of a metasurface is equal to its emissivity, normalized by the blackbody emissivity^64^. Optimizing the absorptivity and emissivity are therefore equivalent.

GAs has already been employed to design broadband angle-independent thermal absorbers^25,65^. Bossard *et al*.^25^, in particular, optimize a metasurface to achieve super-octave absorptivity in the mid-IR regime over a wide ±45° field-of-view. The GA optimized absorber is also polarization independent and its performance has been experimentally validated.

The optimized absorber in our case is a periodic array of building blocks with the configuration depicted in Fig. 7, comprised of a gold back-mirror, a polyimide (PI) substrate, a patterned indium tin oxide (ITO) layer, and a silica superstrate. Polyimide substrates has been previously employed in PV cells, due to their transparency (please see section S-4 in the companion Supplementary Information) and also resilience to high fabrication process temperatures^62,63^. All the dimensions are optimized except for the thickness of the ITO layer, which is considered to be 50 nm. The 15 × 15 ITO pattern has 8-fold symmetry and we optimize one eighth of the pattern. The goal in this design is to achieve broadband, angle- and polarization-independent absorption in the IR regime, and at the same time maintain the transparency of the ITO + SiO_2_ layer, added to the conventional PV cell stack in this design, in the visible spectrum. To achieve this goal, building materials and geometry of the unit-cell are chosen very carefully. More specifically, the 8-fold symmetry of the ITO pattern allows for polarization independence and wide-angle performance of the device. Moreover, ITO acts as an absorptive plasmonic material in the IR regime, and a transparent dielectric in the visible spectrum, with a refractive index close to that of the host silica. Close refractive indexes of ITO and silica causes the incident visible light to pass through the ITO layer with as little disruption as possible. In addition, fused silica also has absorption peaks in IR that contribute to the absorption of the device. More details regarding the material models used in this application are presented in section 4 of the Supplementary Information. In addition to the above-mentioned considerations, we perform a multi-objective optimization to achieve broadband absorption in IR and highest possible transparency in the visible spectrum. The fitness function that we employ in this problem is

8 $$ {\mathcal F} ({\bf{p}})={W}_{{\rm{IR}}}\times {f}_{{\rm{IR}}}({\bf{p}})+{W}_{{\rm{vis}}}\times {f}_{{\rm{vis}}}({\bf{p}})$$

where, *f*_IR_(**p**) and *f*_vis_(**p**) are IR and visible sub-objective functions, respectively, and **p** is a vector representing the parameter domain. In this application, broadband absorption in IR is of significant importance. We define *f*_IR_(**p**) as

9 $${f}_{{\rm{IR}}}({\bf{p}})=\frac{1.0}{({\lambda }_{2}-{\lambda }_{1})}{\int }_{{\lambda }_{1}}^{{\lambda }_{2}}{A}_{{\rm{IR}}}^{2}(\lambda ,{\bf{p}})d\lambda $$

where *A*_IR_(*λ*, **p**) is absorptivity in the IR regime and *λ*_1_ and *λ*_2_ are chosen as 10 μm and 20 μm, respectively. Due to the presence of the back-mirror, which avoids any transmission through the metasurface, *A*_IR_ = 1.0−*R*_IR_, having *R*_IR_ as the reflectance of the array. Transparency of the ITO layer is included in the overall fitness by *f*_vis_(**p**), defined as

10 $${f}_{{\rm{vis}}}({\bf{p}})=\frac{1.0}{N}\sum _{{\lambda }_{i}}{R}_{{\rm{vis}}}^{2}(\lambda ,{\bf{p}})$$

where *R*_vis_(*λ*, **p**) is reflectance of the absorber in visible, ${\lambda ^{\prime} }_{i}s$ are sample wavelengths in the spectrum, and *N* is the number of samples. Since broadband IR absorption is of higher priority than transparency in visible, we use AGA technique to first optimize the metasurface for IR absorption, and then add the visible objective sub-function to the fitness function. More specifically, we first consider IR and visible weights, *W*_IR_ and *W*_vis_ to be 1.0 and 0.0, respectively. After GA has converged to a solution with the best performance in terms of broadband IR absorption, we update the weights in the fitness function to *W*_IR_ = 0.7 and *W*_vis_ = 0.3. Following this adaptive procedure, the final generation in the first stage, with individuals that have superior IR absorption, will be further evolved toward individuals that are both acceptable in terms of IR absorption and also have high transparency in the visible spectrum. More details about the optimization parameters and statistics, together with the GA convergence plot can be found in sections S-5.4.1 and S-4.2 of the Supplementary Information, respectively.

**Figure 8:**
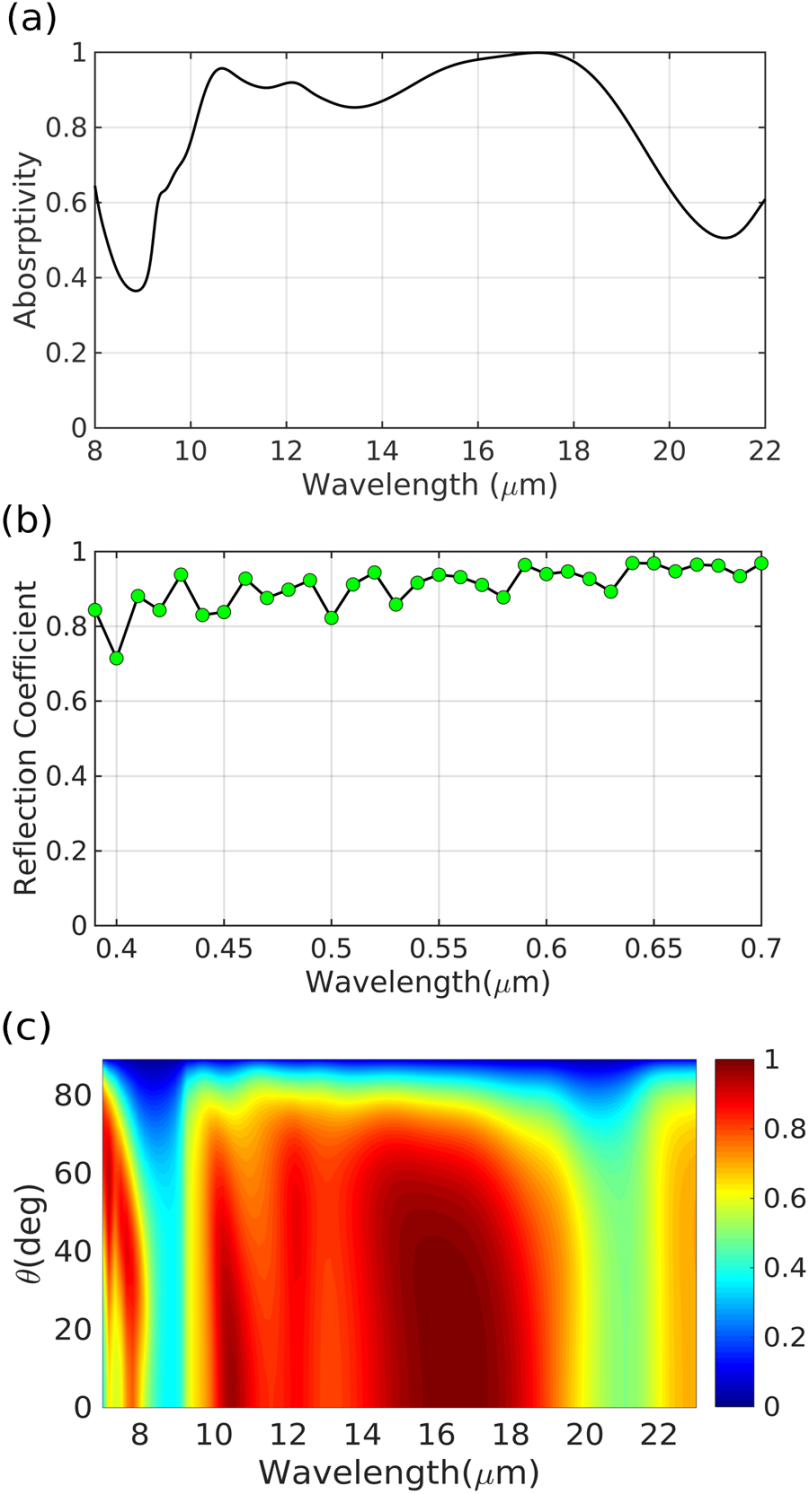
Performance of the optimized dual-band ITO emitter. (**a**) Absorptivity spectrum in the IR regime. (**b**) Reflection spectrum of the metasurface in the visible frequencies. (**c**) Angular absorptivity performance of the optimized emitter.

The Absorptivity spectrum of the optimized metasurface in the IR regime is depicted in Fig. 8(a). As it can be seen in the figure, more than 80% absorption is achieved for nearly one octave in 10–19 μm wavelength range. Furthermore, reflection spectrum of the metasurface is shown in Fig. 8(b). High transparency of the optimized array can be observed in the figure, which is very important to avoid interference with the visible performance of the device. Finally, the angular performance of the metasurface is plotted in Fig. 8(c). It can be observed in the figure that, the metasurface maintains its broadband IR absorption/emission for incidence angles up to about 50°.

### Stochastic Analysis of the Solution Space using surplus data of Genetic Algorithms

The process of optimizing a structure for a target solution using GAs results in more than the solution to a problem. As discussed in the beginning, GAs are based on stochastic analysis of a large set of individual samples, called a generation. The highly ranked individuals in a generation, that best satisfy the objective, are utilized to generate the individuals of the next of generation by crossover and mutation operators. The low ranked individuals are usually discarded. However, one has to note that the process, at no other computational cost, involves solutions of Maxwell’s equations for all the individuals of the generation that can be dynamically stored irrespective of their ranks. A typical solution process for a specific target solves Maxwell’s equations for a few thousand individuals. Such a large pile of data can be utilized as an ingredient to the new-age computational algorithms based on neural networks, such as, deep learning, machine learning, and artificial intelligence, as shown in^48^. Neural network algorithms mimic the functionalities of the neurons of a biological brain to understand, connect, and predict solution to a problem from a large set of examples. Therefore, once solutions are obtained using GA, for example to deliver target phases of 0, *π*/2, *π* and 3*π*/2, the generated surplus data can be utilized to predict the patterns for all the other phase and amplitude targets.

**Figure 9:**
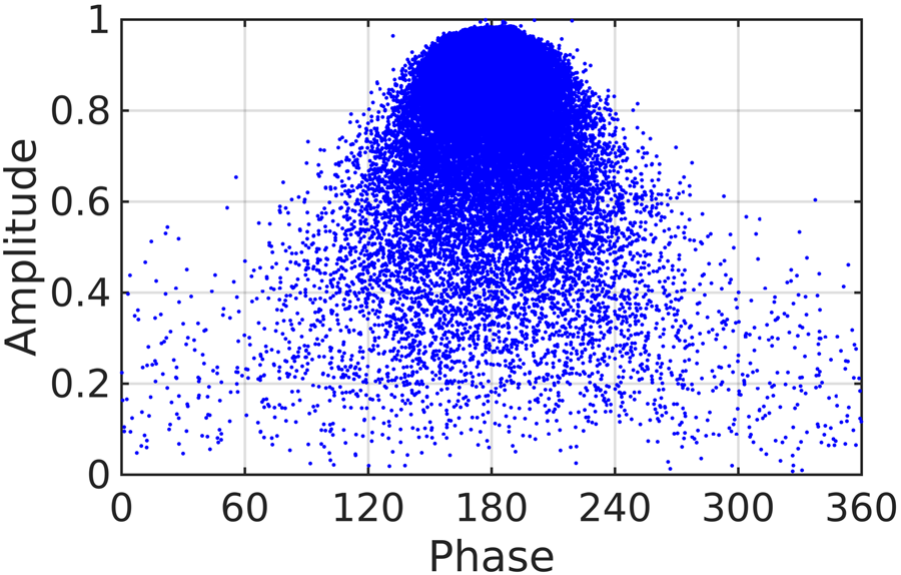
Surplus GA data corresponding to the binary plasmonic metasurface.

Figure 9 presents one such example dataset obtained during the GA optimization of binary-pattern plasmonic metasurface. The binary pattern and the resulted complex reflection amplitudes of each pattern are stored separately during the optimization. To analyze the solution space, the distribution of the phase and amplitude of the reflection coefficient are plotted as shown in Fig. 9. Each dot represents the result of an individual (or binary pattern). The grid lines help to empirically sense the relative density of the distribution of dots. The high density of dots in the region with phase 180° and amplitude 1.0 are the samples where the specular reflection from the back mirror is dominant.

Many inferences can be immediately made by observing the dataset. For example,

(i) An ideal choice of a family of building blocks is the one that uniformly distributes the dots in the amplitude-phase plot. Such a uniform distribution provides all possible choices required in arbitrary shaped wavefront engineering, based on holography. Figure 9 depicts that the family of binary patterns is a close example of such case. Conventional metasurfaces, built on the basis of building blocks of canonical shapes, will result in only few dots depending on the geometrical parameters.
(ii) In addition, for applications such as bending or focusing light, a phase distribution ranging from 0 to 360° with a uniform high amplitude is preferred. The uniformity in amplitude reduces the contribution to parasitic side-lobes of the radiation pattern. The dataset immediately aids in identifying patterns with full phase range and a number of choices of uniform amplitudes.

**Figure 10:**
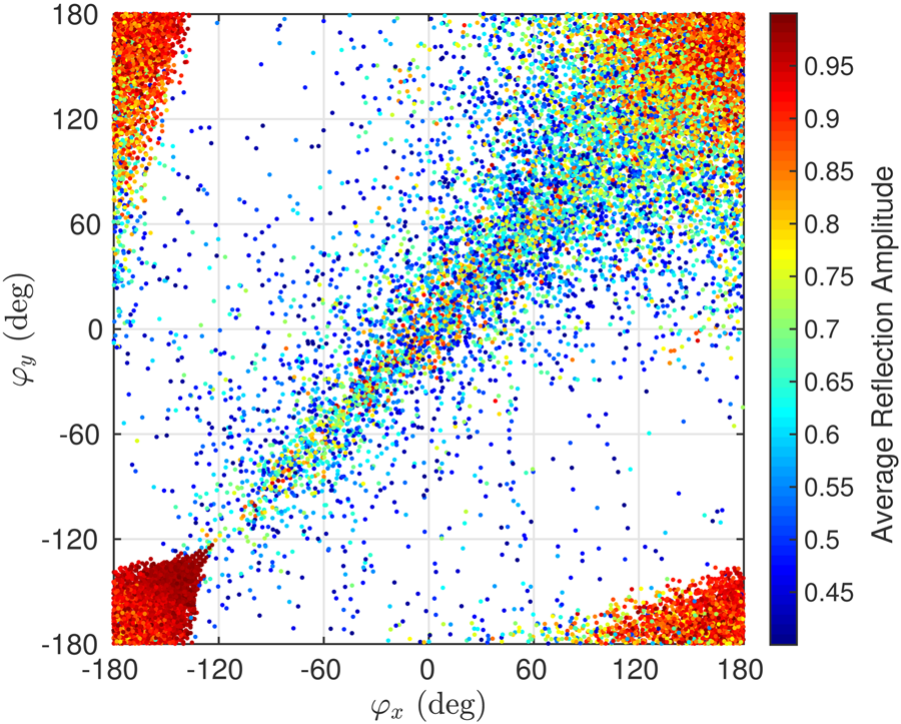
GA data corresponding to the birefringent all-dielectric metasurface.

A second dataset example, shown in Fig. 10, is obtained from the birefringent all-dielectric metasurface optimization. Each dot in the figure represents the phase of *x-* and *y-*polarized field components of an individual of a generation, computed during the GA optimization. The color of each dot represent the product of amplitudes of *x-* and *y-*polarized field components. Note that the product *ab* for (0 < *a* < 1) and (0 < *b* < 1) is maximum only when *a* = *b* = *1*. Similar to the previous case, uniform distribution of points throughout the grid is expected for complete independent control of two polarizations on a single platform. For beam scanning application, requiring independent control on bending direction of each polarization, presence of at least one dot in each box with high and uniform amplitude is expected. Figure 10 clearly shows that such a point can be found in each box, with at least an amplitude of 0.4 for each polarization. The corresponding binary patterns of points chosen along the horizontal (vertical) axis will manipulate *x-*(*y-*)polarized component of the field. Binary patterns corresponding to the points chosen along the diagonal will manipulate both polarizations similarly.

**Figure 11:**
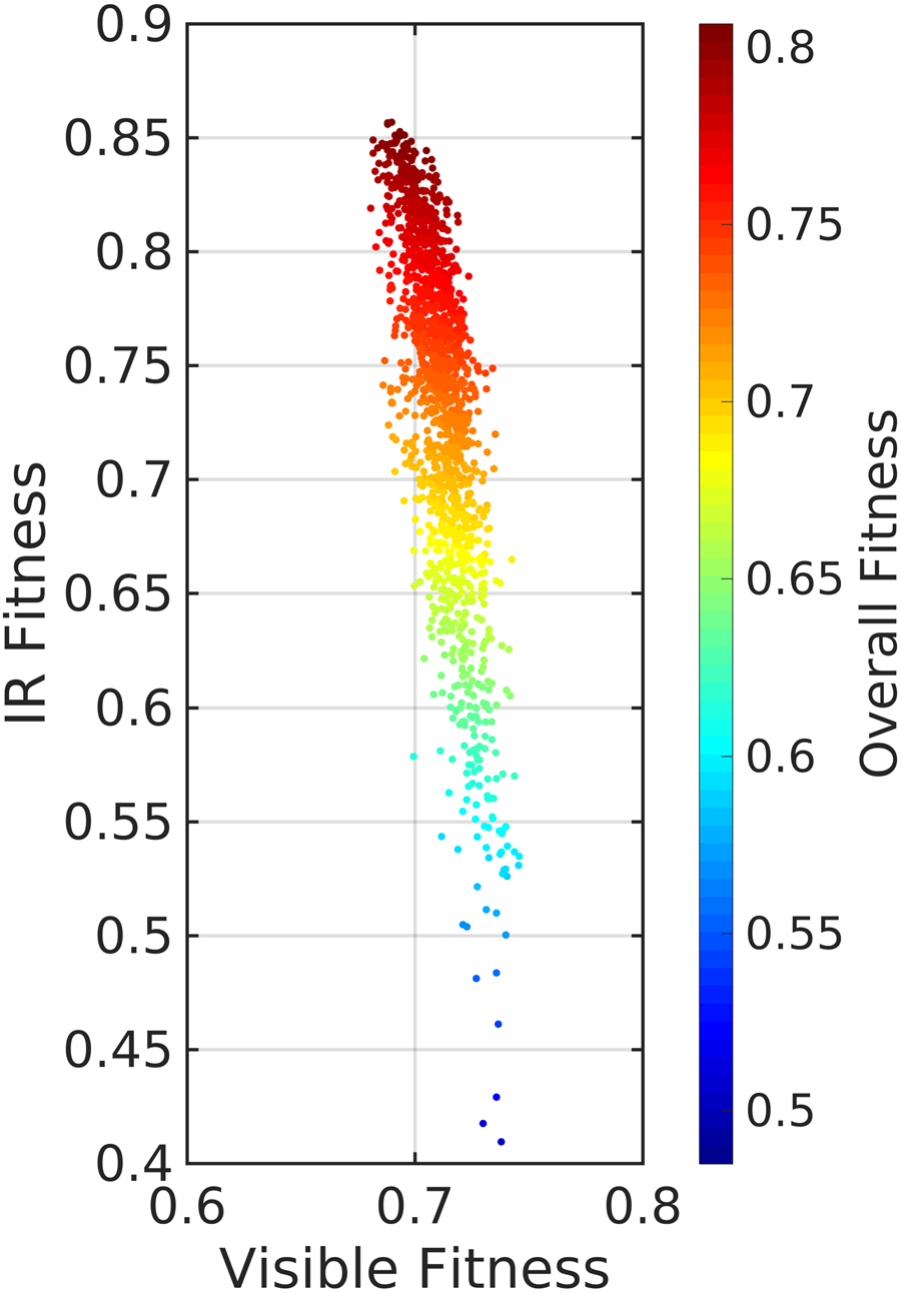
GA data corresponding to the visible-transparent IR-absorbing/emitting metasurface.

Figure 11 represent a dataset obtained from the visible-transparent IR-emitting metasurface. Making an appropriate use of the AGA technique, we collected the fitness values of the unit-cell for both IR and visible spectra for a large number of samples, analyzed during the optimization. Each dot represent a single solved unit-cell. The negative inclination of the distribution of the points shows the challenge of the problem, that the best optimized structure (with higher fitness) for transparency in the visible regime presents the lowest absorption performance at IR, and vice versa. Utilizing the dataset, one may choose a pattern with desired balance between IR absorbency and visible transparency.

## Conclusion

In summary, we have introduced adaptive genetic algorithm (AGA), as a powerful evolutionary optimization technique, capable of handling complex design problems and large parameter spaces in the field of optical metasurfaces. To demonstrate the advantages of the AGA technique, compared to the traditional design methods, we have solved four novel problems of interest to the optics community. We have successfully optimized a binary-pattern plasmonic reflectarray for beam-steering applications. By means of adaptive optimization, we have considered both accurate retardation phase and high reflection amplitude of the constitutive unit-cells as sub-objectives with different levels of priority in the fitness function. Moreover, this device offers higher tolerance to fabrication imperfections, compared to canonical plasmonic nano-antennas. Next, we have optimized the whole array of an asymmetrical leaky-wave antenna (LWA), intended to radiate TE and TM excitation waveguide modes to arbitrarily chosen directions. Optimization of such devices is impossible with local optimization methods, due to prohibitively large number of parameters, and the achieved functionality cannot be obtained from conventional periodic LWAs. Asymmetrical geometry of a compact birefringent all-dielectric metasurface has then been optimized, which offers finer lateral resolution of elements compared to the conventional high-index nano-antennas. Finally, We have demonstrated a visible-transparent infrared (IR) emitting/absorbing device, incorporating a sophisticated combination of building materials, including ITO and fused silica, for solar-cell cooling applications. We have considered both broadband, wide-angle absorption in IR and transparency in visible as sub-objectives in the fitness function, and given higher priority to the IR absorption. All the physical parameters have been optimized to achieve a solution that can be incorporated in solar-cells to enable radiative cooling, while having minimal interference with the visible functionality of the cell. Although we have not considered more than two sub-objectives in our presented designs, we believe that the AGA cycle, in the flowchart of Fig. 1, can be readily continued after two iterations to add more than two objectives to the fitness function of eq. 2. It is well known, and has been observed in our optimizations, that, after a sufficient number of generations, there would be a substantial number of individuals in the population with fitness values very close to the highest fitness value. Updating the fitness function can therefore be thought of as discriminating in favor of some of the fittest individuals, based on the added sub-objective. In that sense, continuing the AGA cycle after two iterations (considering more than two sub-objectives) seems totally plausible. As a path to a future work, we have shown that large datasets, generated as by-products of GAs, can be used as ingredients for new-age computational algorithms, such as, machine learning and deep learning, enabling a shift of paradigm from computing to predicting targeted solutions. Applying the new concepts demonstrated in this paper can help one to handle challenging problems of high current interest in the field of optical metasurfaces, and pave the way for fulfilling the great potential of these devices to substitute bulky refractive optical components.

## Methods

We have linked our full-wave in-house developed highly parallel FDTD (PFDTD) solver^66^ with an open-source genetic algorithm (GA) driver^67,68^ to optimize the applications presented in this paper. The GA driver and our GPU-enabled FDTD solver are developed in Fortran and OpenCL, respectively. Our hybrid PFDTD-GA solver allows for fast, wide-band, and accurate characterization of metasurface structures, which is of extreme importance for any successful optimization task. Furthermore, we have solved our optimization problems on a computer cluster equipped with many GPU-equipped nodes, enabling us to run several optimization tasks in parallel, giving rise to speed-up factors equal to the number of GPU nodes. This has been a significant advantage in this work, especially for designing binary-pattern plasmonic, and birefringent all-dielectric matasurfaces, where all of the retardation phases or phase combinations have been optimized simultaneously. Taking advantage of the broadband FDTD analysis, we have been able to characterize the visible-transparent IR-emitting/absorbing metasurface in the whole frequency band of interest with one single run. Also, PFDTD solver enables us to optimize large arrays, such as the presented dual-beam aperiodic LWA, thanks to its fast GPU-computing capabilities.

The angular response of the visible-transparent IR-emitting/absorbing metasurface is obtained by an in-house built Rigorous Coupled-Wave Analysis (RCWA) solver^69^. RCWA solver is a tailor-made solver for periodic arrays. RCWA computes the complex amplitude coefficients of TE and TM polarized diffracted waves, defined by the grating equation, by matching the boundary conditions at each interface. A total of 1250 diffraction orders has been used for computing the angular dependence of absorption shown in Fig. 8(c). The absorption is defined as *A* = 1 − (*R*_TE_ + *R*_TM_)/2, Where *R*_TE_ and *R*_TM_ are sums of reflection coefficients, corresponding to TE and TM components of all diffraction orders, respectively.

## Electronic supplementary material

Supplementary Information
