# Supplementary material for: Adaptive Genetic Algorithm for Optical Metasurfaces Design
Source: Sci Rep. 2018 Jul 23;8:11040. doi: 10.1038/s41598-018-29275-z (PMC6056425; doi:10.1038/s41598-018-29275-z)
Supplement: Supplementary file 1 — Supplementary Information [file 41598_2018_29275_MOESM1_ESM.pdf]

Supplementary Information

attached to

"Adaptive Genetic Algorithm for Optical Metasurfaces Design"

Scientific Reports:

Samad Jafar-Zanjani, Sandeep Inampudi, and Hossein Mosallaei

S-1 An Introduction to Genetic Algorithm

In this section we briefly review the main concepts of genetic algorithm (GA)–more generally, evolutionary optimization. This is meant for readers who might not be familiar with GAs and is included for the sake of self-sufficiency. Another objective of this section is to put the GA parameters presented in section S-5 into perspective. The interested reader is, however, urged to consult standard GA literature, such as ref.[5], for more information.

There are two main categorises of optimization techniques: local and global optimizers[8]. Local optimizers are tightly bound to the solution domain and take advantage of initial guesses, which makes their converge to a solution relatively fast. However, due to this close bound, they impose some limitations such as continuity to the solution domain and the objective function. There is also a high chance for local optimizers to become stuck in a local extremum close to the initial guess, instead of the optimal global solution. Global optimizers on the other hand, are mostly independent of the solution domain and therefore do not impose the mentioned constraints on the optimization problems. They have, however, slower convergence rates compared to local optimizers. Problems of optimizing metasurfaces with complex geometries, such as binary patterns in this paper, involve discrete solution domains, and discontinuous or non-differentiable objective functions. Among global optimization techniques, GAs are the most suitable methods to handle such problems.

GAs are stochastic search optimizers that are based on the concepts of evolution and natural selection. GAs are global optimizers, which makes their convergence a little bit slow, but, enables them to find the near-optimal solution of the problems at hand. Yet in the optimization problems encountered in the field of photonics and specially metasurfaces, due to the demanding design goals and considerable fabrication costs, finding the best possible solution is of significant importance and overshadows the slower convergence rates.

Major concepts of GA are presented in fig.S-1. GAs usually work with encoded versions of *individuals* (prototype solutions) of an optimization problem. Each individual is characterized by a set of parameters involved in the problem, for instance, geometrical dimensions or material composition, and can therefore be represented by a vector $\mathbf{p} = (p_1, p_2, ..., p_n)$ in the n-dimensional parameter space. In the case of a binary encoding, parameters in vector $\mathbf{p}$ are converted to bit strings, called *genes*, and combination of genes form the corresponding *chromosome* of an individual[7], $\mathbf{q} = (q_1, q_2, ..., q_n)$, where each q_i is a bit string of length m (therefore, $\mathbf{q}$ consists of $n \times m$ bits). Binary encoding is illustrated in fig.S-1(a). The connection between a GA and the optimization

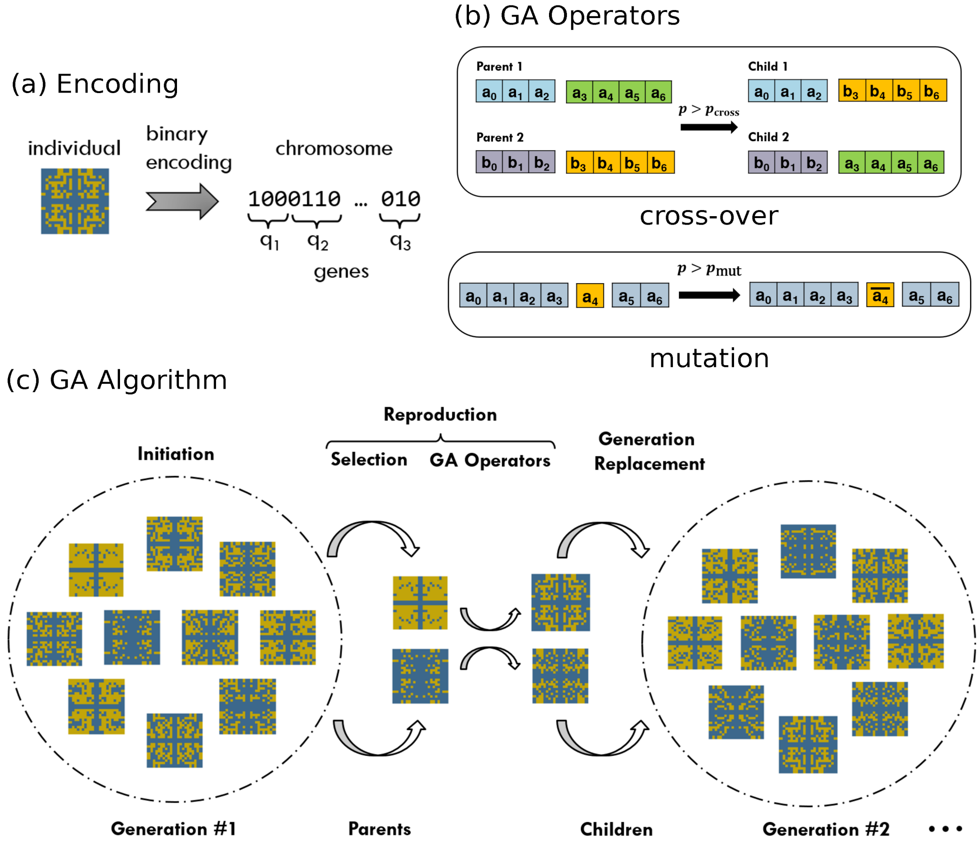

Fig. S-1: Genetic algorithm concepts. (a) Binary encoding of individuals. (b) GA operators. (c) Three phases of a GA: initiation, reproduction, and generation replacement.

problem is provided by *fitness functions*, $\mathcal{F}(\mathbf{p})$. A fitness function assigns a figure of merit or a measure of goodness[8] to a specific individual, which is usually a positive number and called *fitness*. The goal is to find an individual with maximum possible fitness, or the optimal solution. It should be noted that, since GAs work with chromosomes, there should be a decoding step ($\mathbf{p} \rightarrow \mathbf{q}$) before evaluation of the fitness function.

Genetic algorithms consist of three main phases[8], namely, i. *initiation*, ii. *reproduction*, and iii. *generation replacement*. In the *initiation* phase, a population of randomly generated chromosomes, an initial generation, is formed. This initial generation is then evolved based on the fitness function to the proceeding generations, including more *fit* individuals, through reproduction and generation replacement processes. *Reproduction* process, takes advantage of GA operators, including, selection, cross-over, and mutation. One of the most successful selection strategies is *tournament selection*[5, 8], in which a sub-population of individuals are randomly selected from the current GA generation. These selected individuals then compete based on their fitness and the one with the highest fitness is selected. These selected *parents* (members of the current generation) then go through reproduction process to generate members of the next generation, or *children*. The most important GA operators in the reproduction phase are *cross-over* and *mutation*, depicted in fig.S-1(b). The standard cross-over (recombination) operator takes two parent chromosomes as input and generates two child chromosomes. In order to do so, a random position is selected along the chromosome string, and the segments before and after the selected positions are exchange between the parents (see upper panel of fig.S-1(b)), with a probability of p_{cross}. On the other hand, mutation

operator selects a random element in the chromosome string, with a probability of p_{mutation}, and inverts it. The purpose of cross-over and mutation operators are recombining the genes between members of a population, and searching the parts of the solution domain that are not represented by the current members of the population, respectively, with the hope of generating more fit children. Finally, in the *generation replacement* of GA, the current generation of parents is replaced with the new generation of children, generated as explained above. It should be mentioned that, in this paper, population size, N_{pop}, has been kept constant for all the generations in an optimization. Number of generations is represented by N_{gen}. Also, we have used an *elitist* strategy[8] to ensure monotonic increase in the highest fitness values of consecutive generations. In the generation replacement phase, the individual with highest fitness value, in the current generation, is transferred to the next generation to realize such elitism. A schematic of three main GA phases is presented in fig.S-1(c). We have used unit-cell patterns from one of the applications presented in the companion paper, the binary plasmonic metasurface for beam-steering, to illustrate initiation, reproduction, and generation replacement phases of GA.

Flowchart of a conventional genetic algorithm is depicted in fig.S-2. The algorithm starts by initiation of the first generation with N_{pop} random chromosomes. Then, it proceed by assigning fitness values to the members of the current population, by decoding the individuals from chromosome space to parameter space[8], and evaluating the fitness function. If the *stop criteria* (for instance, specific number of generations or a threshold for the highest fitness value) is met, the algorithm will terminated and the *near-optimal* solution is obtained. Otherwise, the *selection* strategy, *tournament selection* is this paper, is utilized to select eligible parents form current generation for recombination and mutation, with probabilities of p_{cross} and p_{mutation}, respectively. The current generation is then replace with the next generation in the *generation replacement* phase, taking advantage of *elitism*, as explained above.

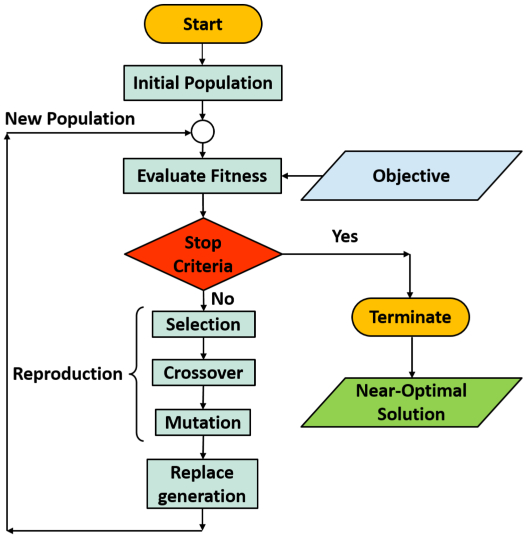

Fig. S-2: Flowchart of a conventional genetic algorithm.

S-2 Design Procedure for the Binary Pattern Reflectarray Application

It is a well-known concept that, for a 2-D beam-steering reflectarray, the distribution of the reflection phase-delay over the aperture of the array should have the following form[4]:

$$\psi(x, y) = k_0 \sin(\theta_0) \left[x \cos(\varphi_0) + y \sin(\varphi_0)\right] = k_x x + k_y y \tag{S-1}$$

where k_0 is the free-space wavenumber and (θ_0, φ_0) is the targeted direction of the reflected beam. It is worth mentioning that, the phase distribution of eq.S-1 is periodic both in x and y with periodicities of $\Lambda_x = 2\pi/k_x$ and $\Lambda_y = 2\pi/k_y$, respectively. The common practice in designing reflectarrays is to quantize the phase-delay distribution is eq.S-1 to a few levels and realize those phase levels by the metasurface elements. As an example, in section 3.1 the companion paper, we have targeted (30°,45°) as the direction of the reflected beam, and also considered 8 levels of quantization. The resulting quantized phase-delay distribution is plotted in fig.S-3(a), for 3 periods of the phase-delay distribution in both x and y directions.

In order to realized the above-mentioned quantized phase distribution by a reflectarray, one needs to take advantage of metasuface elements with retardation phases equal to the quantization levels. In this work, we have employed the AGA technique to design 8 binary plasmonic unit-cells to realized the phase distribution of fig.S-3(a). Each 8×8 block of unit-cells is called a super-cell, with Λ_x and Λ_y dimensions in x and y directions, respectively. The resulting phase distribution over the aperture of the reflectarray is calculated by the FDTD method and depicted in fig.S-3(b). Periodic boundary condition is used to terminate the solution domain in the lateral directions.

By means of near-field to far-field transformation, the reflection pattern of the plasmonic reflectarray is calculated for a 10×10 configuration of super-cells (see fig.3(a) of the companion paper). The resulting directivity pattern is depicted in fig.S-4. The mainlobe is magnified in the inset, showing a 3dB beamwidth of 2°.

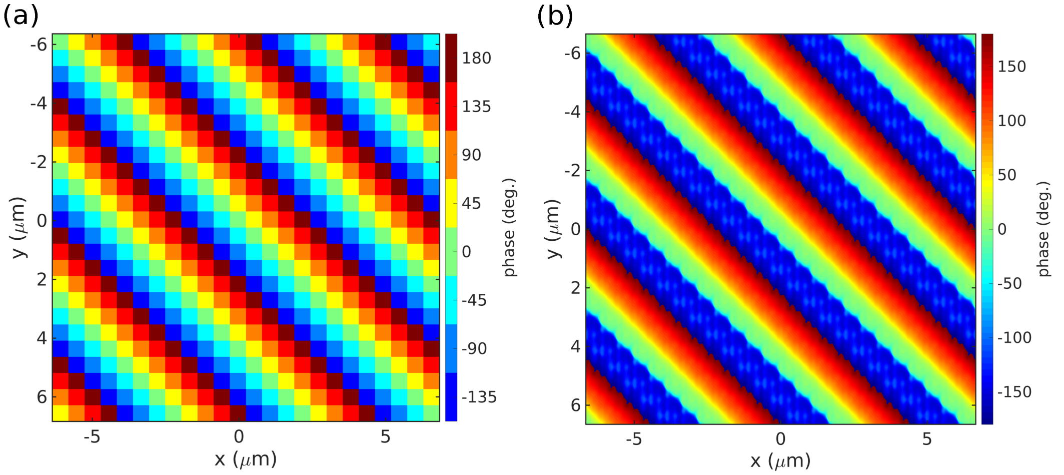

Fig. S-3: Phase-delay distribution of a beam-steering metasurface for reflecting the normally incident beam to (θ_0, φ_0)=(30°,45°). (a) Ideal Phase distribution, quantized to 8 levels and plotted for 3 periods in both x and y directions. (b) Realized phase distribution of a mosaic of AGA-designed plasmonic unit-cells, calculated by the FDTD method. Periodic boundary condition is utilized to terminate the solution domain in the lateral directions.

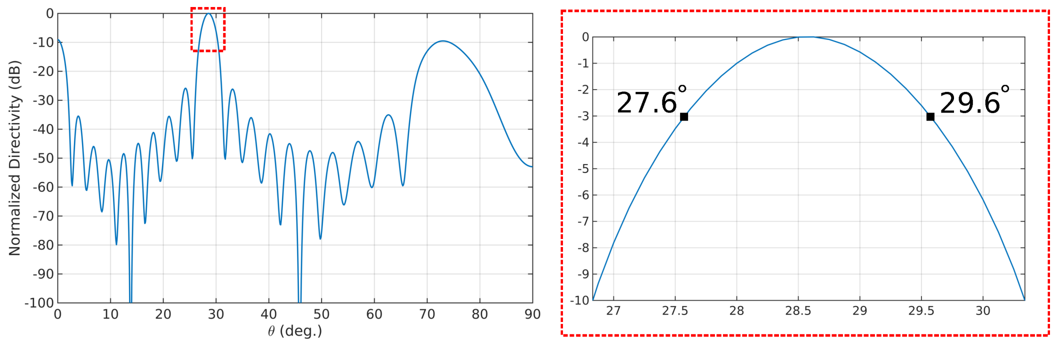

Fig. S-4: E-plane directivity pattern of the binary pattern plasmonic reflectarray designed by the AGA technique to reflect an normally incident beam to (θ_0,φ_0)=(30°,45°). Mainlobe is magnified in the right-hand inset plot, and shows a 3dB beamwidth of 2°.

S-3 More details regarding the Dual-Beam LWA application

S-3.1 Conventionl Leaky-Wave Antennas

Leaky wave antennas (LWAs) operate on the principle of diffraction. Conventional LWAs use periodic ridges (gratings) inscribed on top of slab waveguides to couple the guided modes of the slab to radiative modes of free-space. The periodicity (Λ) of the grating is chosen such that one of the diffraction orders (usually -1 indexed diffraction order) of the waveguide mode is directed towards a desired angle (θ_0). Assuming the modal index of the waveguide mode is given by (β), the periodicity can be expressed as $\Lambda = \lambda_0/(\beta/k_0 - sin\theta_0)$. Note that β depends on the polarization of the waveguide mode. Accordingly, for a given Λ, waveguide modes of each polarization get radiated to different angles. Figure S-5(a) presents an example case where a LWA is excited by two line-sources with y and z polarizations. The dimensional parameters, including the periodicity of the grating, are given in the caption. It can be seen in fig.S-5(b) that the y and z polarized line-sources, excite transverse-electric (TE) and transverse-magnetic (TM) waveguide modes on the slab waveguide, respectively. The TE excitation mode gets radiated by the LWA to 34° and the TM mode to $-22°$, as it can be seen in fig.S-5(c)-(f).

Figure S-6 shows the possible (fixed) beaming angles for TE and TM polarized waveguides modes in dielectric slab of the LWA shown above as a function of periodicity of the grating. The angles are computed using the formula $\theta = \arcsin(\beta/k_0 - \lambda_0/p)$. As seen, the angular difference between the two polarizations is fixed by the periodicity of an LWA (and almost constant). The GA optimization methodology employed in this paper allows us to choose unrestricted beaming angles.

S-3.2 Another example of an optimized Dual-Beam LWA

In order to further investigate the capability of GAs to design an aperiodic dual-beam LWA introduced in section 3.2 of the companion paper, we have solved another set of targeted radiation directions, namely, +45° and -45° for y and x components of the electric field, respectively. The results are presented in fig.S-7. It can be observed in the figure that the optimized LWA has mainlobes very close to the targeted radiation directions.

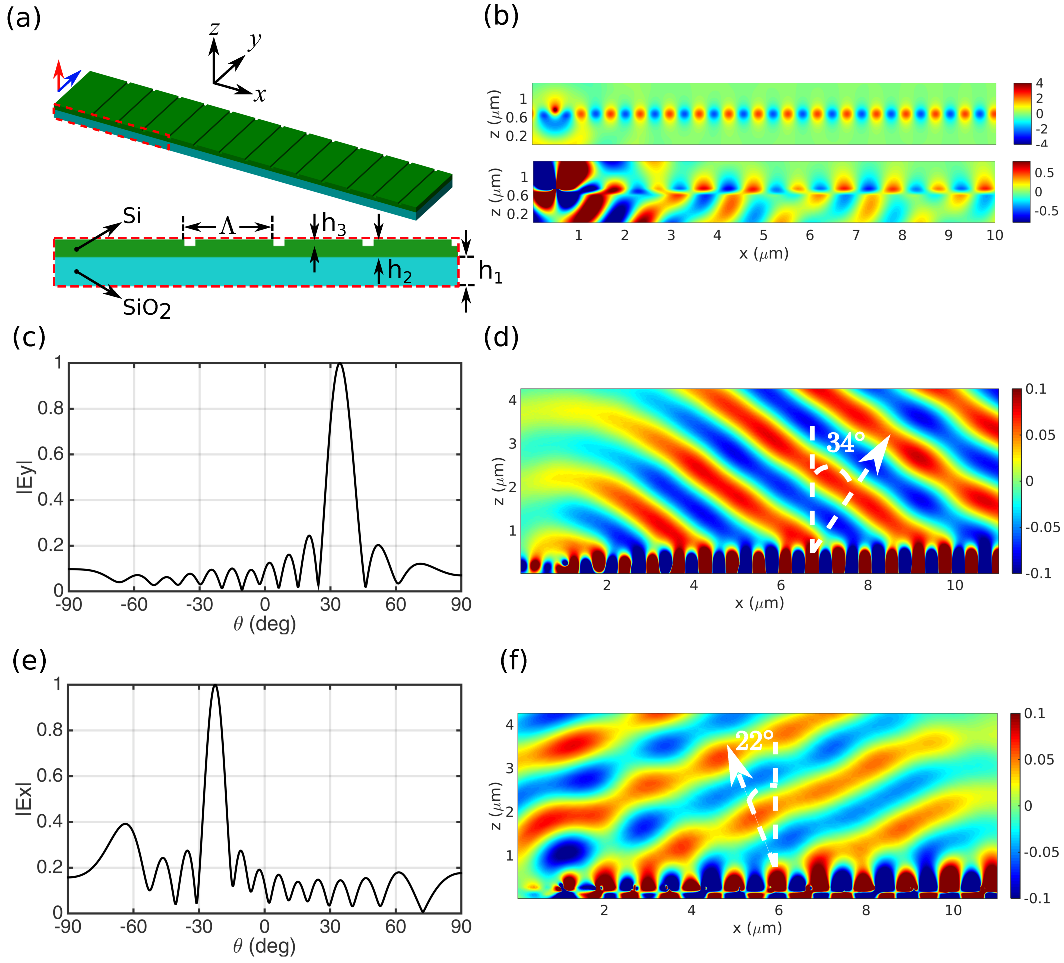

Fig. S-5: (a) Schematic of a conventional LWA. Parameters: $h_1 = \infty$ (half-space), $h_2 = 160\,\text{nm}$, $h_3 = 60\,\text{nm}$, $\Lambda = 800\,\text{nm} = 0.52\lambda_0$ (periodicity). Operation frequency is 193.5 THz ($\lambda_0 = 1.55\,\mu\text{m}$). (b) The upper image shows the TE, and the lower image shows the TM excitation modes, respectively. (c) Far-field radiation pattern of the y-component of the electric field. (d) Near-field plot, showing the y-component of the electric field. (e) Far-field radiation pattern of the x-component of the electric field. (f) Near-field plot, showing the x-component of the electric field.

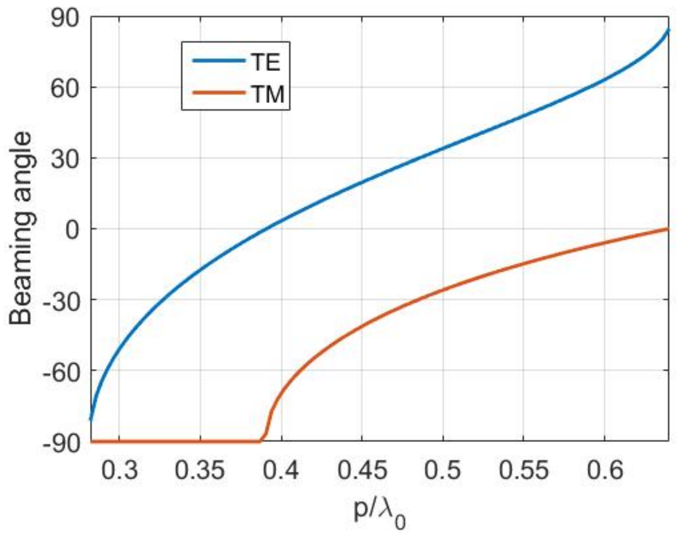

Fig. S-6: Beaming angles of a periodic LWA for TE and TM polarizations

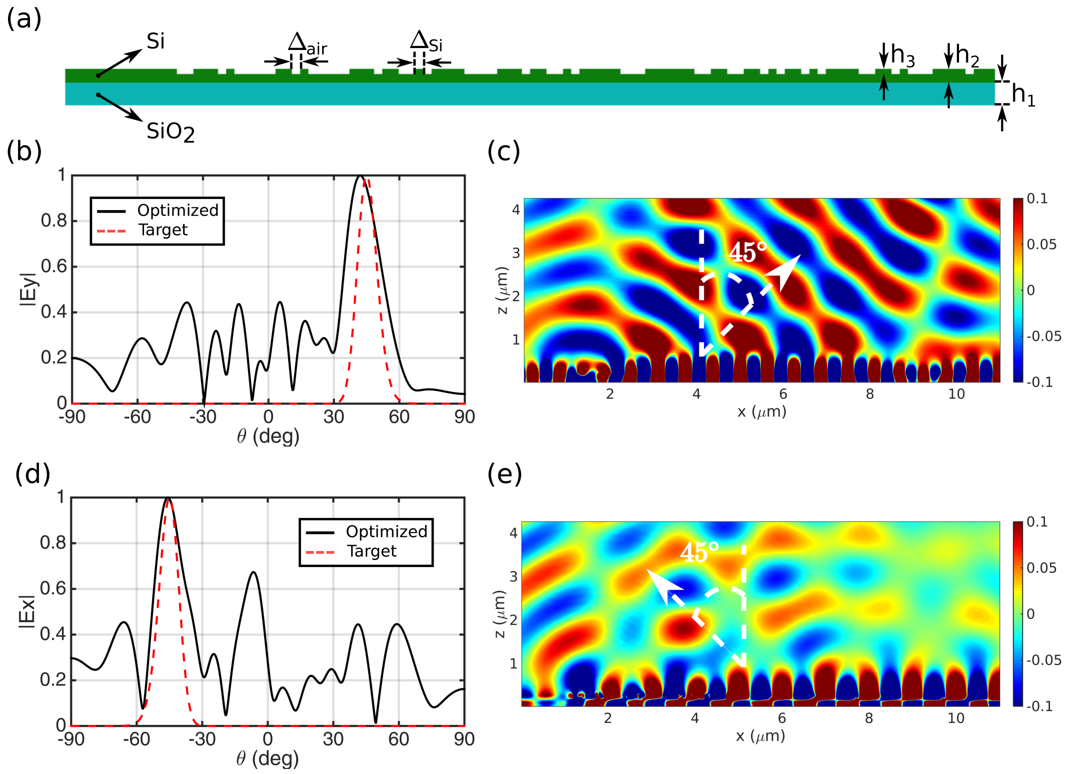

Fig. S-7: Another example of designing a dual-beam LWA antenna to radiate TE and TM excitation waveguide modes to +45° and -45°, respectively. (a) Side view of the optimized antenna. Parameters: $\Delta_{\text{air}} = \Delta_{\text{Si}} = 100\,\text{nm}$, $h_1 = \infty$ (half-space), $h_2 = 160\,\text{nm}$, $h_3 = 60\,\text{nm}$. Operation frequency is 193.5 THz ($\lambda_0 = 1.55\,\mu\text{m}$). (b) Far-field radiation pattern of the y-component of the electric field. (c) Near-field plot, showing the y-component of the electric field. (d) Far-field radiation pattern of the x-component of the electric field. (e) Near-field plot, showing the x-component of the electric field.

S-4 Material Models for Visible-Transparent Infrared-Emitting Metasurface

We have used the FDTD method to accurately characterize the metasurface unit-cell, corresponding to visible-transparent IR-emitting application in section 3.4 of the companion paper, in the IR regime. In order to incorporate material dispersion in FDTD, a dispersion model should be fit to the wavelength dependent permittivity data. Drude dispersion model is employed for silver (Ag) and Lorentz model for polyimide (PI), fused silica(SiO_2), and indium tin oxide(ITO).
Drude dispersion model can be written as follows[12]:

$$\varepsilon(\omega) = \varepsilon_\infty - \frac{\omega_p^2}{\omega^2 - j\omega\gamma_p} \tag{S-2}$$

where ε_∞ is the relative permittivity at infinite frequency, ω_p is the frequency of the Drude pole, and γ_p is the inverse of the pole relaxation time[12]. Also, Lorentz model has the following form:

$$\varepsilon(\omega) = \varepsilon_\infty + \sum_{p=1}^{N_{\text{poles}}} \frac{\Delta\varepsilon_p \omega_p^2}{\omega_p^2 + 2j\omega\delta_p - \omega^2} \tag{S-3}$$

where N_{poles} is the number of Lorentz pole pairs, $\Delta\varepsilon_p$ is the change in the relative permittivity due to the p'th pole pair, ω_p is the undamped frequency of the p'th pole pair, and δ_p is the damping coefficient[12].

We have employed $\varepsilon_\infty = 5.0$, $\omega_p = 1.3666\text{E}{+}16$, and $\gamma_p = 2.7332\text{E}{+}13$ as Drude model parameters of Ag[11]. Moreover, parameters represented in table S-1 are used in the Lorentz models fit to PI, ITO, and SiO_2[9] in IR. Relative permittivities of the materials of table S-1 are also plotted in fig.S-8.

Transfer matrix method is utilized in the visible wavelengths to calculate the reflection coefficient of the absorber. Unlike the IR regime, in visible, we directly use the permittivity data in our calculations at uniformly distributed sample points throughout the spectrum. Due to the deeply sub-wavelength dimensions of the ITO pattern and smooth variation of the permittivities throughout the visible spectrum, the effective medium approximation yields accurate results for the reflection coefficient of the absorber in visible. Validity of this approximation is verified by comparison with full-wave simulations. Furthermore, effective medium approximation makes the AGA optimizations very fast, without that much loss of accuracy, and is crucial for this application. The permittivity data for Ag[1], PI, ITO, and SiO_2[9, 10], along with the corresponding sample wavelengths are given in table S-2. It can be seen in the table that ITO acts as a low-loss dielectric in visible, and permittivity contrast between SiO_2 host and ITO pattern is small. Relative permittivities of table S-2 are plotted versus wavelength in fig.S-9.

Table S-1: Material parameters of the visible-transparent infrared-emitting metasurface in the IR regime.

Parameters	PI	ITO	SiO_2
N_{poles}	8	8	6
ε_∞	2.390	3.260	2.100
$\Delta\varepsilon_1$	3.5160E-04	6.3126E+00	8.3933E-02
ω_1	3.3718E+14	6.0854E+13	1.5059E+14
δ_1	8.4191E+11	7.1630E+12	6.0289E+12
$\Delta\varepsilon_2$	1.2252E-02	3.2428E+00	1.0259E-01
ω_2	3.2593E+14	5.0193E+13	1.1700E+14
δ_2	2.5051E+12	2.5535E+13	1.1657E+13
$\Delta\varepsilon_3$	2.8420E-03	8.9968E+00	4.6482E-01
ω_3	2.5880E+14	8.9262E+13	1.9818E+14
δ_3	1.0821E+12	1.8922E+13	5.5005E+12
$\Delta\varepsilon_4$	1.1229E-02	2.2683E+01	1.7428E-01
ω_4	2.8378E+14	7.1276E+13	2.0420E+14
δ_4	1.1654E+12	1.2858E+13	2.7162E+12
$\Delta\varepsilon_5$	3.5763E-03	1.2162E+01	5.1118E-01
ω_5	2.6060E+14	8.3174E+14	8.2156E+13
δ_5	1.2496E+12	1.6254E+15	4.1992E+12
$\Delta\varepsilon_6$	2.2196E-02	4.9259E+01	4.7437E-01
ω_6	1.5837E+14	5.2865E+13	8.6521E+13
δ_6	3.4209E+13	0.0000E+00	2.2622E+12
$\Delta\varepsilon_7$	4.3780E-02	4.8393E+01	–
ω_7	2.3468E+14	5.5672E+13	–
δ_7	6.3026E+12	9.9676E+12	–
$\Delta\varepsilon_8$	4.8878E-02	1.0000E+00	–
ω_8	2.2691E+14	1.6047E+15	–
δ_8	6.5010E+12	6.6574E+13	–

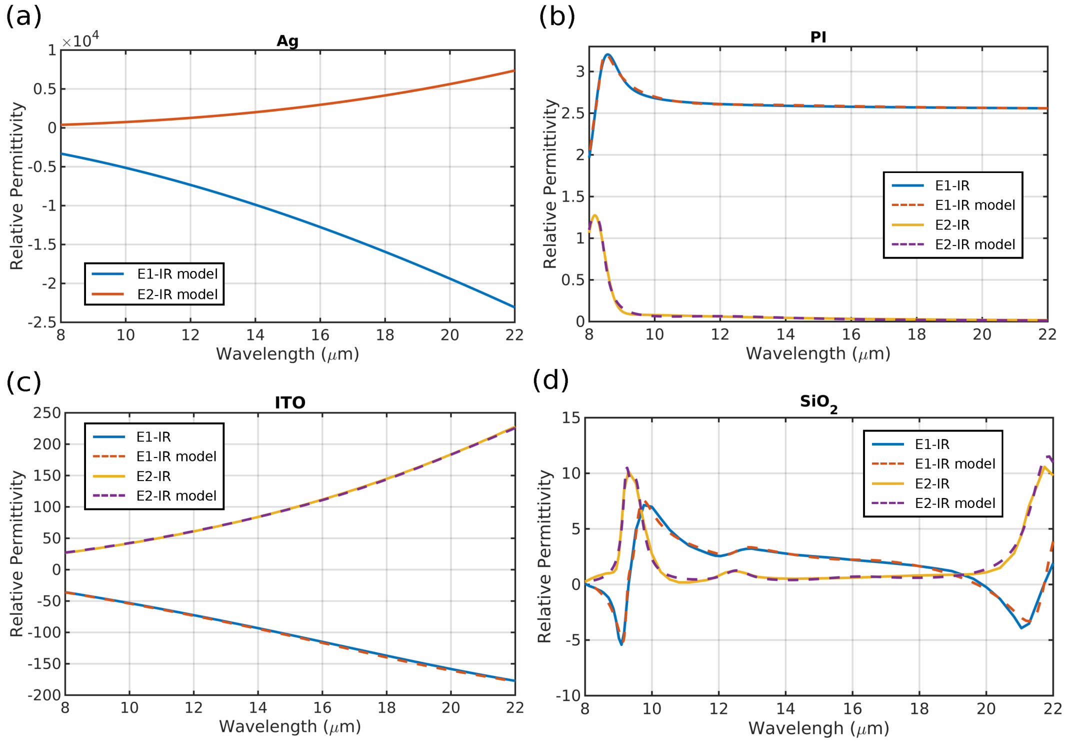

Fig. S-8: Relative permittivities of (a) Ag, (b) PI, (c) ITO, and (d) SiO_2 versus wavelength in the IR regime, corresponding to material models in table S-1. E1 and E2, in the legends of the graphs, represent the real and imaginary parts of the dielectric function, respectively.

Table S-2: Material parameters of the visible-transparent infrared-emitting metasurface in the visible spectrum.

Wavelength (nm)	Ag	PI	ITO	SiO_2
390	-3.8414 + 0.91138i	2.6781	4.3529 + 0.21945i	2.2056
400	-4.393 + 0.96746i	2.6622	4.2808 + 0.2989i	2.2025
410	-4.9612 + 1.0153i	2.6468	4.2639 + 0.27327i	2.1996
420	-5.5185 + 1.0618i	2.6323	4.172 + 0.25339i	2.1969
430	-6.0572 + 1.1097i	2.6194	4.1142 + 0.28507i	2.1944
440	-6.582 + 1.159i	2.6072	4.112 + 0.28014i	2.1921
450	-7.098 + 1.2098i	2.5963	4.0789 + 0.22208i	2.1899
460	-7.61 + 1.2622i	2.586	3.9986 + 0.17851i	2.1879
470	-8.1231 + 1.3163i	2.5767	3.9027 + 0.17827i	2.186
480	-8.6421 + 1.3721i	2.5679	3.841 + 0.23627i	2.1843
490	-9.1721 + 1.4298i	2.5596	3.8691 + 0.24955i	2.1826
500	-9.7179 + 1.4893i	2.5521	3.8264 + 0.21396i	2.1811
510	-10.282 + 1.5506i	2.5451	3.8105 + 0.23424i	2.1796
520	-10.863 + 1.6133i	2.5385	3.78 + 0.1931i	2.1783
530	-11.461 + 1.6772i	2.5322	3.7984 + 0.23147i	2.177
540	-12.074 + 1.7419i	2.5264	3.7465 + 0.13044i	2.1757
550	-12.702 + 1.807i	2.5211	3.6567 + 0.15536i	2.1746
560	-13.342 + 1.8723i	2.5159	3.679 + 0.20355i	2.1735
570	-13.995 + 1.9375i	2.511	3.6908 + 0.17384i	2.1725
580	-14.659 + 2.0022i	2.5066	3.6792 + 0.12096i	2.1715
590	-15.334 + 2.066i	2.5022	3.6272 + 0.085426i	2.1706
600	-16.017 + 2.1287i	2.4982	3.5722 + 0.079412i	2.1697
610	-16.708 + 2.19i	2.4943	3.53 + 0.098807i	2.1689
620	-17.407 + 2.2494i	2.4906	3.532 + 0.12472i	2.1681
630	-18.112 + 2.3068i	2.4873	3.5449 + 0.096707i	2.1673
640	-18.823 + 2.3622i	2.4838	3.5191 + 0.062447i	2.1666
650	-19.541 + 2.4157i	2.4808	3.4843 + 0.044543i	2.1659
660	-20.265 + 2.4673i	2.4779	3.4514 + 0.035171i	2.1653
670	-20.997 + 2.5172i	2.4751	3.4215 + 0.029682i	2.1646
680	-21.736 + 2.5655i	2.4724	3.394 + 0.02613i	2.164
690	-22.482 + 2.6122i	2.4697	3.3683 + 0.023655i	2.1635
700	-23.237 + 2.6574i	2.4674	3.3442 + 0.021834i	2.1629

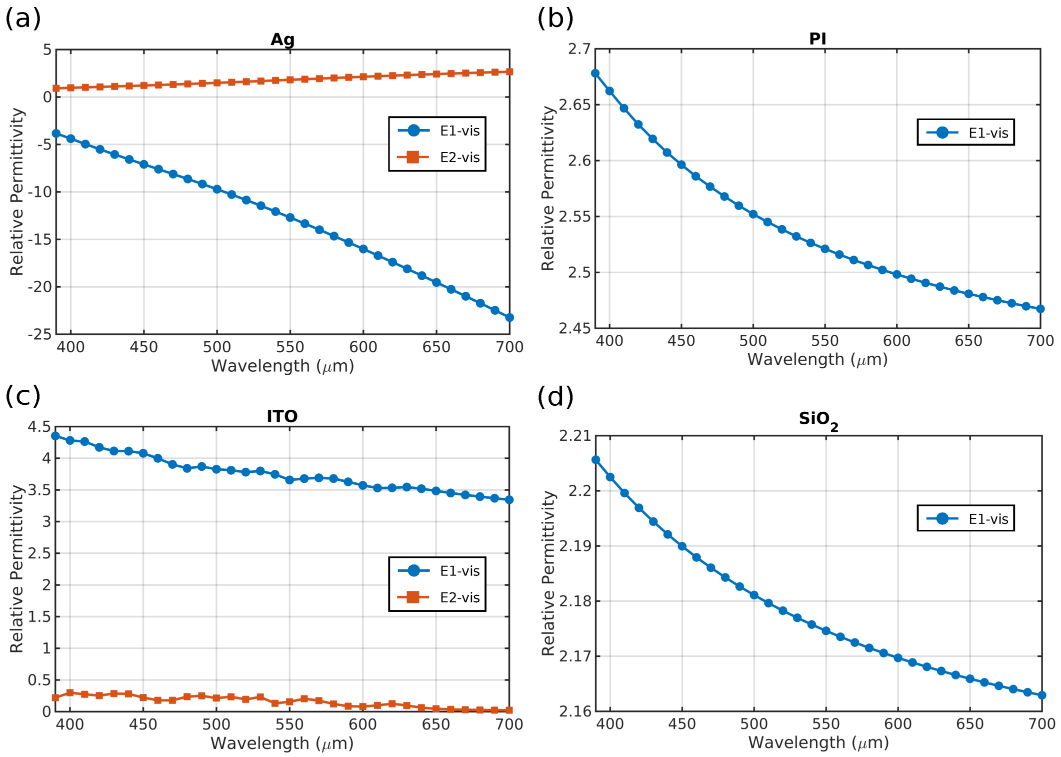

Fig. S-9: Relative permittivity plots versus wavelength in the visible spectrum for (a) Ag, (b) PI, (c) ITO, and (d) SiO_2, corresponding to table S-2. E1 and E2, in the legends of the graphs, represent the real-part and imaginary-part of the dielectric function, respectively.

S-5 More Details Regarding the Performed Optimizations in the Companion Paper

In this section, we present GA parameters and statistics corresponding to the applications presented in section 3 (Results and Discussion) of the companion paper. An introduction to GAs along with a brief discussion of its main parameters is given in section S-1 of this supporting document. Table S-3 summarize the parameters that will be presented in this section with short descriptions. There are some well known rules regarding the choice of GA parameters, as follows. A rule of thumb is suggested in ref.[3] for the choice of the population size, N_{pop} as:

$$N_{\mathrm{pop}} = \mathcal{O}\left[(l/k)\chi^{k}\right]. \tag{S-4}$$

where l is the chromosome length, k, effectively, is the average number of bits per parameter, and $\chi = 2$ for binary encoding. In our case $l = N_{\mathrm{par}}$, and $k = 1$. Therefore, $N_{\mathrm{pop}} = \mathcal{O}(2N_{\mathrm{par}})$. However, we have been able to achieve good convergence in most cases with $N_{\mathrm{pop}} \approx N_{\mathrm{par}}$. Higher value for N_{pop} leads to more genetic diversity[8], and therefore, faster convergence. it also implies, however, larger fitness evaluation time for each generation. From our experience, number of generations should be chosen as $N_{\mathrm{gen}} = \mathcal{O}(N_{\mathrm{pop}})$. For multi-objective optimization, we have split N_{gen} equally among the AGA stages. Probability of crossover, p_{cross}, is typically chosen in 0.6-0.9 range, with an optimal value of 0.7 for most of the problems[8]. Higher values of p_{cross} results in faster search in the chromosome domain. Probability of mutation, p_{mut}, should be chosen small, usually in 0.01-0.1 range[8].

Table S-3: Summary of GA parameters.

Parameters	Description
N_{par}	Number of parameters
N_{pop}	Population size
N_{gen}	Number of generations
p_{cross}	Probability of cross-over
p_{mut}	Probability of mutation
T_{ind}	Simulation time for a single individual
T_{opt}	Total Optimization time
GPU Memory	Required GPU memory

S-5.1 Binary Pattern Reflectarray Metasurface for Beam-Steering

S-5.1.1 Fitness Function

The fitness function for this problem is defined as

$$\begin{aligned}\mathcal{F}(\mathbf{p}) &= W_{\varphi} \times \exp[-6.0\,(\varphi(\mathbf{p}) - \varphi_{\mathrm{target}})^2] \\ &+ W_a \times \exp[-4.0\,(1.0 - A(\mathbf{p}))^2]\end{aligned} \tag{S-5}$$

First and second exponential terms in eq.S-5 represent phase and amplitude objective sub-functions, respectively, and are plotted in fig.S-10. The constant factors in the exponents of the Gaussian sub-functions (-6.0 and -4.0 for phase and amplitude, respectively) are chosen carefully, based on

the physical considerations and goals of the problem. The phase objective function has been chosen to have a sharp peak, giving rise to accurate optimized phases and fast GA convergence. Since one cannot expect perfect reflection amplitudes close to 1.0 for many retardation phases in this application, as will be discussed in section S-5.1.3, we have chosen a smoother Gaussian function as the amplitude objective. Using this amplitude objective function, one can expect high amplitude fitnesses even for amplitudes around 0.7-0.8.

S-5.1.2 Optimization Parameters and Statistics

GA parameters and statistics corresponding to the binary pattern reflectarray metasurface application in section 3.1 of the companion paper are presented in table S-4. Taking advantage of a GPU-enabled parallel FDTD (PFDTD) solver[6], we have been able to solve each GA individual (see section S-1) is 16 s. We have also taken advantage of a computer cluster equipped with GPU nodes to optimize all the 8 distinct phase-delays simultaneously, resulting in a speed-up factor of 8. Although it takes about 45 h to design this metasurface, with a specific set of goals, we have solved 80,000 distinct unit-cell patterns in the course of the optimization. It has been discussed in section 4 of the paper, that this available surplus data can be used to design metasurfaces with alternative goals, without further computational cost.

Table S-4: GA parameters used for the binary pattern reflectarray metasurface in section 3.1 of the companion paper.

Parameters	Value
N_{par}	100
N_{pop}	100
N_{gen}	100
p_{cross}	0.7
p_{mut}	0.02
T_{ind}	16 s
T_{opt}	44.4 h
GPU Memory	17.6 MB

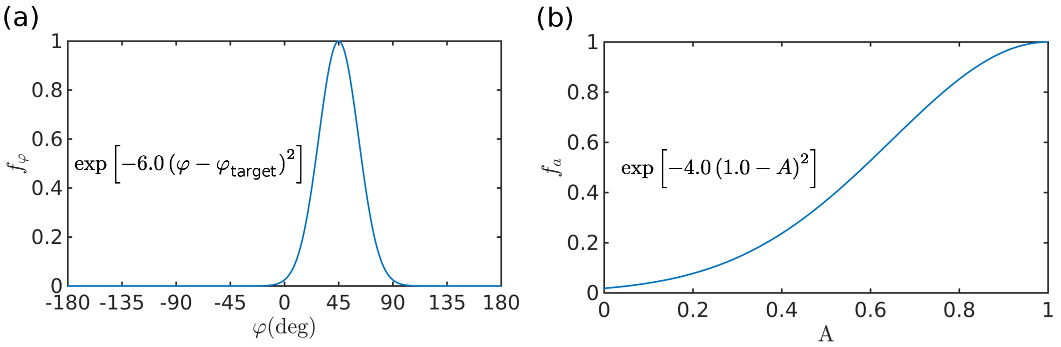

Fig. S-10: Objective sub-functions corresponding to the binary pattern reflectarray metasurface application in section 3.1 of the companion paper. (a) Phase objective function ($\varphi_{\mathrm{terget}} = 45°$). (b) Amplitude objective function.

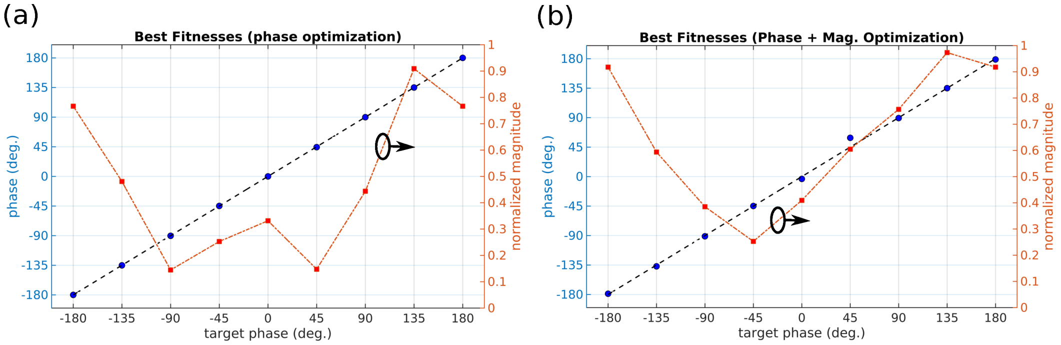

Fig. S-11: Optimized values of retardation phase and reflection magnitude for two stages of the adaptive optimization. The horizontal axes show 8 levels of the targeted phase-delays. (a) Phase optimization: near-optimal phase-delay values are achieved. (b) Phase and magnitude optimization: reflection magnitudes are significantly improved and retardation phases remain in an acceptable range from the targeted values.

S-5.1.3 GA Convergence Plots

It has been mentioned in section 3.1 of the paper, that we have employed a two-stage adaptive procedure to optimize the unit-cells of the binary pattern metasurface. In the first stage, we just optimize phase-delay of the unit-cells, and in the second stage we optimize both phase-delay and magnitude of the reflection coefficient. Results of the adaptive optimization are presented in fig.S-11. As it can be seen in fig.S-11 (a), at the end of the first stage, optimal values for all of the targeted phase-delays have been achieved. In the second stage, we consider a small weight for the magnitude objective sub-function, in eq.S-5. Consequently, as it can be observed in fig.S-11 (b), we have been able to improve the reflection magnitudes for almost all of the targeted phase-delays, and also the optimized phases do not deviate too much from the optimal values achieved in the first phase of the optimization, and remain in a acceptable range from the targeted values.

Furthermore, GA convergence plots, showing maximum fitness values in each GA generation versus generation index are plotted in fig.S-12. A discontinuity can be observed in all the convergence plots at the 51st generation, where the fitness function is changed based on the adaptive optimization. It can also be seen in fig.S-12 that, for almost all of the targeted phase-delays, an improvement in the overall fitness is achieved at the end of the second phase of the adaptive optimization (except for ϕ = -45°).

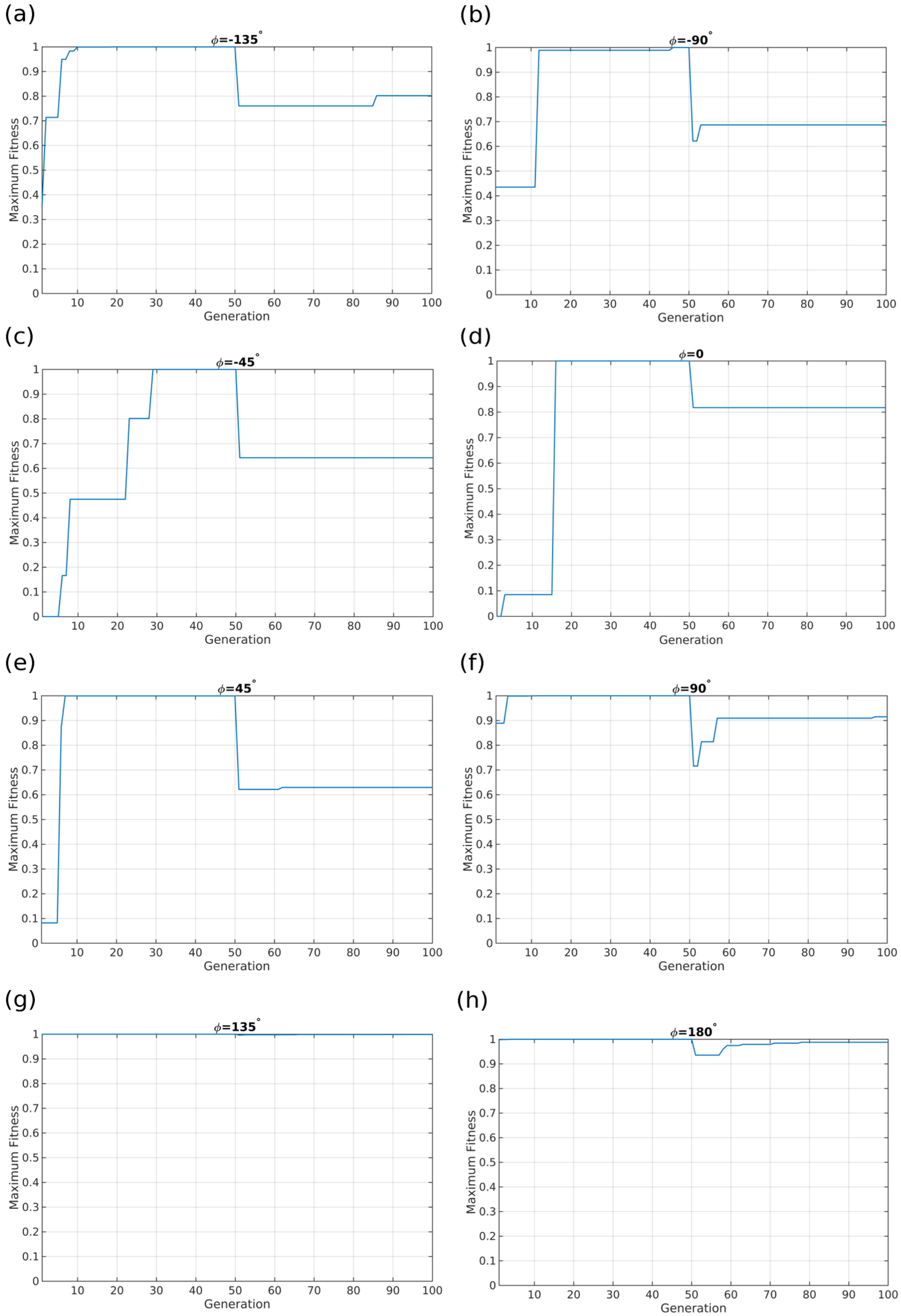

Fig. S-12: (a)-(h) GA convergence plots for the binary pattern reflectarray metasurface in section 3.1 of the companion paper, showing maximum fitness value of each GA generation versus the generation index. Fitness values change between 0 and 1.0. Each of the 8 panels represents one specific retardation phase targeted for the unit-cell, as specified in the title of each plot (see section S-2 for more details). The sudden changes in the maximum fitness values, at 51st generation, are due to the adaptive optimization.

S-5.2 Dual-Beam Aperiodic Optical Leaky Wave Antenna

S-5.2.1 Fitness Function

Cross-correlation of two arbitrary functions $v(t)$ and $w(t)$, that can in general be complex-valued, is defined as [2]

$$R_{vw}(\tau) = (v \star w)(\tau) \triangleq \int_{-\infty}^{+\infty} v^*(t)w(t+\tau)dt \tag{S-6}$$

The cross-correlation operator can be normalized as follows:

$$\hat{R}_{vw}(\tau) \triangleq \frac{R_{vw}(\tau)}{\sqrt{R_{vv}(0)R_{ww}(0)}} \tag{S-7}$$

so that it returns 1.0 if both function are the same and values less than 1.0 if they are different.

For this application, the fitness function is defined as

$$\mathcal{F}(\mathbf{p}) = W_y \times f_y(\mathbf{p}) + W_x \times f_x(\mathbf{p}) \tag{S-8}$$

where f_y and f_x have the same form. For instance, f_y can be written as

$$f_y(\mathbf{p}) = \left(|E_y|(\mathbf{p}) \star |E_y|^{\text{target}}\right)(\theta = 0) \tag{S-9}$$

In other words, $f_y(\mathbf{p})$ is the zero-lag normalized cross-correlation of $|E_y|$ and $|E_y|^{\text{target}}$.

S-5.2.2 Optimization Parameters and Statistics

GA parameters and statistics corresponding to the dual-beam aperiodic LWA, presented in section 3.2 of the companion paper, are presented in table S-5.

Table S-5: GA parameters used for dual-beam aperiodic optical LWA in section 3.2 of the companion paper.

Parameters	Value
N_{par}	100
N_{pop}	100
N_{gen}	50
p_{cross}	0.7
p_{mut}	0.02
T_{ind}	26 s
T_{opt}	36.1 h
GPU Memory	22 MB

S-5.2.3 GA Convergence Plot

GA convergence plot for the dual-beam LWA example in section 3.2 of the paper is presented in fig.S-13. Monotonic increase in the maximum overall fitness values of GA generations can be seen in the figure. We have also presented maximum values of f_x and f_y objective sub-functions in the same figure. It should be mentioned that these maximum fitness values do not necessarily correspond to the maximum overall fitness values. For instance, an individual in the generation with maximum f_x fitness, do not necessarily possess the maximum overall fitness.

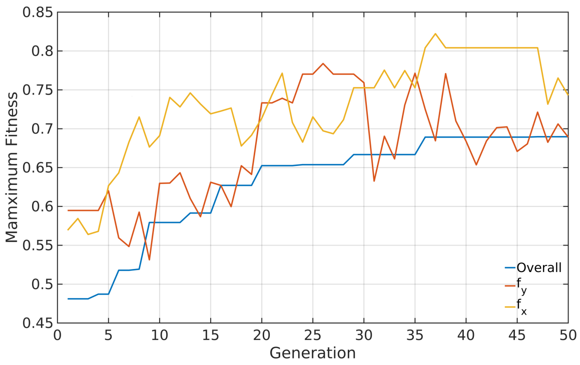

Fig. S-13: GA convergence plot, corresponding to the dual-beam aperiodic LWA in section 3.2 of the companion paper, showing the maximum fitness value of each GA generation versus the generation index. Fitness values change between 0 and 1.0. The fitness function consists of two terms, associated with x and y components of the radiated field (f_x and f_y, respectively). The maximum values of f_x and f_y versus generation index are also plotted in the figure.

S-5.3 Compact Birefringent All-Dielectric Metasurface

S-5.3.1 Fitness Function

We have employed the following fitness function

$$\mathcal{F}(\mathbf{p}) = W_x \times f_x(\mathbf{p}) + W_y \times f_y(\mathbf{p}) \tag{S-10}$$

for the compact birefringent all-dielectric metasurface in section 3.3 of the companion paper. Identical objective sub-functions f_x and f_y correspond to x and y components of the reflected field, respectively, and are plotted in fig.S-14. We have chosen the constant factor -6.0 in the Gaussian sub-functions carefully to assure sharp peaks at the targeted phase-delays, and therefore accurate optimized phase-delay for each component of the reflected field.

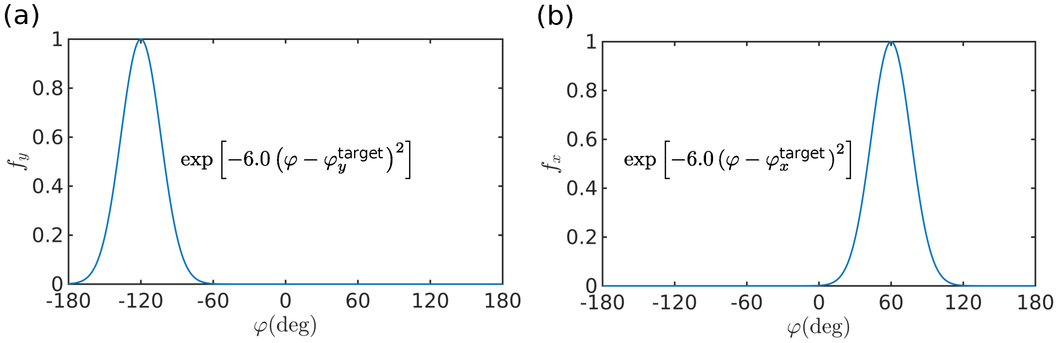

Fig. S-14: Objective sub-functions corresponding to the compact birefringent all-dielectric metasurface in section 3.3 of the companion paper. (a) Objective function corresponding to the y component of the reflected field, f_y (for $\varphi_y^{\text{terget}} = -120°$). (b) Objective function corresponding to the x component of the reflected field, f_x (for $\varphi_y^{\text{terget}} = 60°$).

S-5.3.2 Optimization Parameters and Statistics

Table S-6 presents the GA parameters and statistics corresponding to the compact birefringent all-dielectric metasurface in section 3.3 of the companion paper. It is worth mentioning that, taking advantage of a computer cluster equipped with GPU-enabled nodes, we have been able to optimize all of the 26 distinct phase-delay combinations, corresponding to perpendicular components of the reflected field, simultaneously. This has provided us with a significant advantage, as, we have been able to design the whole birefringent metasurface in an optimization time equal to that of a single phase-delay combination (a speed-up factor of 26). Moreover, 32,500 GA individuals have been solved in about 27 h of optimization time, giving rise to a pool of surplus data that can be employed for other design goals, without further computational cost (see section 4 of the companion paper).

Table S-6: GA parameters used for the compact birefringent all-dielectric metasurface in section 3.3 of the companion paper.

Parameters	Value
N_{par}	25
N_{pop}	50
N_{gen}	25
p_{cross}	0.7
p_{mut}	0.02
T_{ind}	77 s
T_{opt}	26.7 h
GPU Memory	37.8 MB

S-5.3.3 GA Convergence Plots

GA convergence plots for this application are also presented in fig.S-15. Each panel corresponds to one of the 26 phase combinations (φ_x,φ_y), for x and y components of the reflected beam, as specified in the title of each panel. As it can be seen in the figure, for most of the phase combinations, GA has been able to converge to the targeted values.

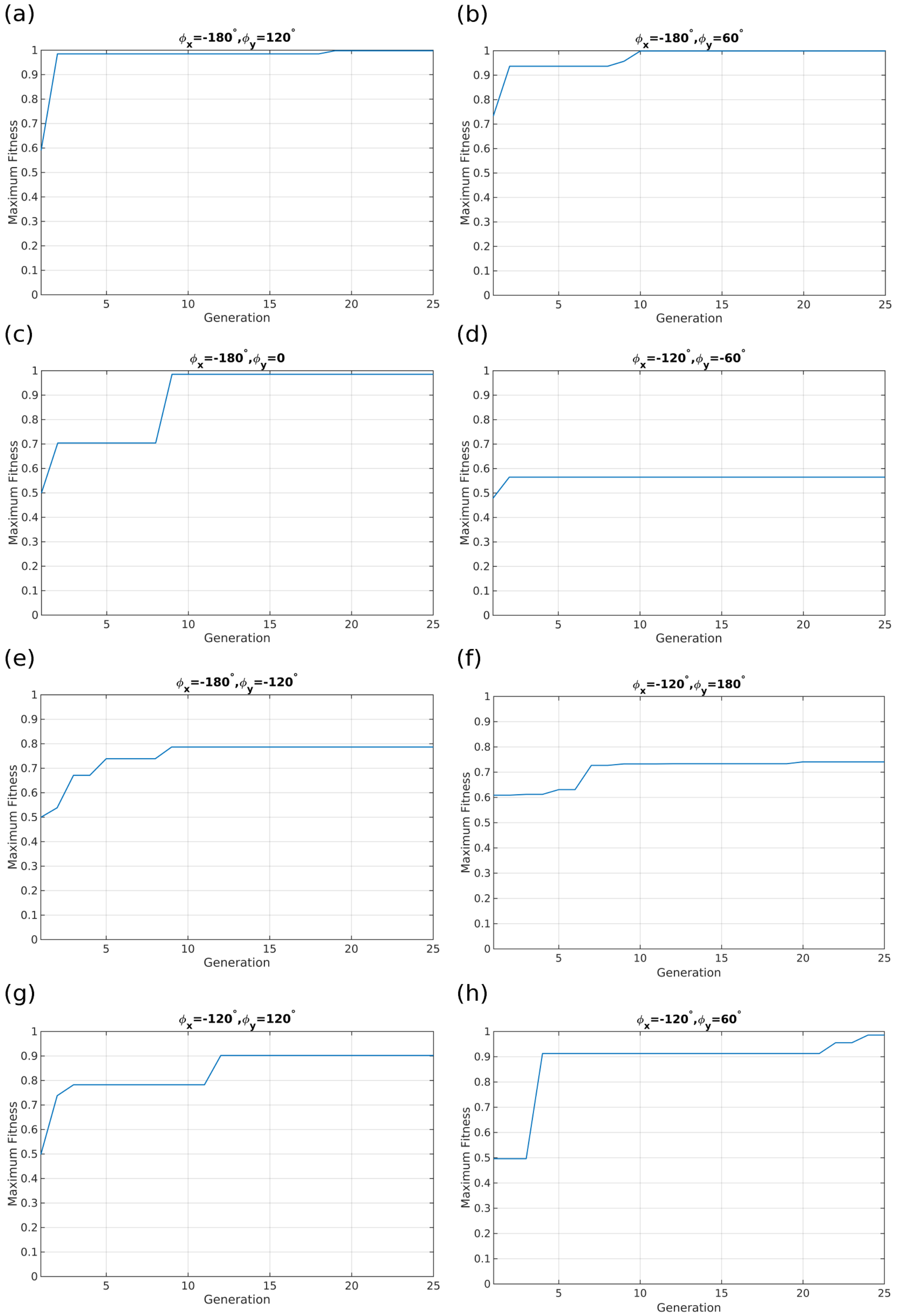

(a)
ϕ_x=-180°,ϕ_y=120°
Maximum Fitness
Generation
(b)
ϕ_x=-180°,ϕ_y=60°
Maximum Fitness
Generation
(c)
ϕ_x=-180°,ϕ_y=0
Maximum Fitness
Generation
(d)
ϕ_x=-120°,ϕ_y=-60°
Maximum Fitness
Generation
(e)
ϕ_x=-180°,ϕ_y=-120°
Maximum Fitness
Generation
(f)
ϕ_x=-120°,ϕ_y=180°
Maximum Fitness
Generation
(g)
ϕ_x=-120°,ϕ_y=120°
Maximum Fitness
Generation
(h)
ϕ_x=-120°,ϕ_y=60°
Maximum Fitness
Generation

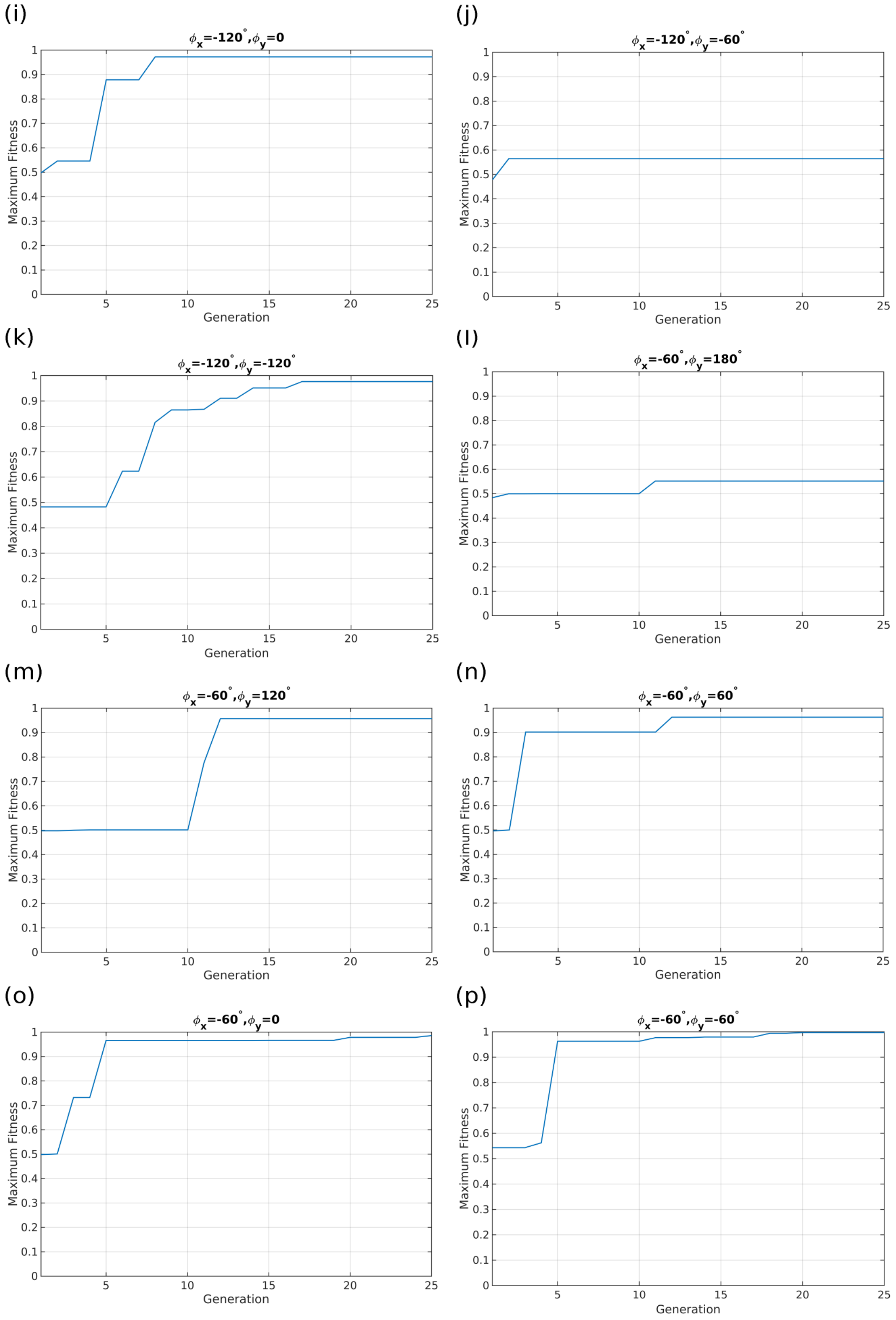

(i)
ϕx=-120°,ϕy=0
(j)
ϕx=-120°,ϕy=-60°
(k)
ϕx=-120°,ϕy=-120°
(l)
ϕx=-60°,ϕy=180°
(m)
ϕx=-60°,ϕy=120°
(n)
ϕx=-60°,ϕy=60°
(o)
ϕx=-60°,ϕy=0
(p)
ϕx=-60°,ϕy=-60°
Maximum Fitness
Generation

(q)

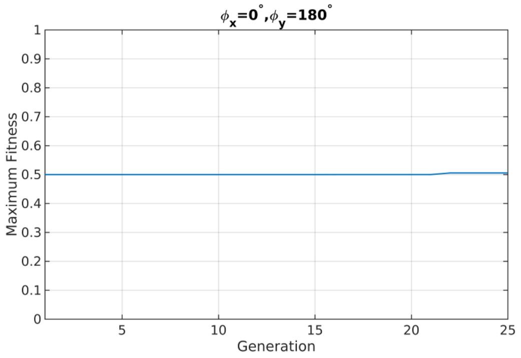
ϕ_x=0°,ϕ_y=180°
Maximum Fitness
Generation

(r)

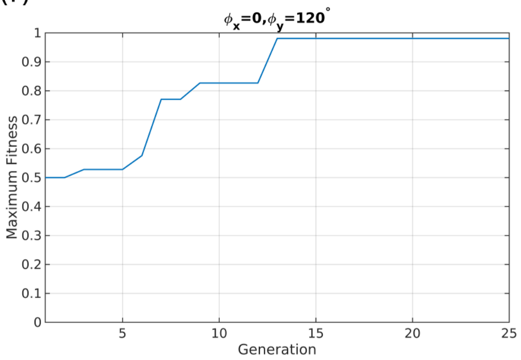
ϕ_x=0,ϕ_y=120°
Maximum Fitness
Generation

(s)

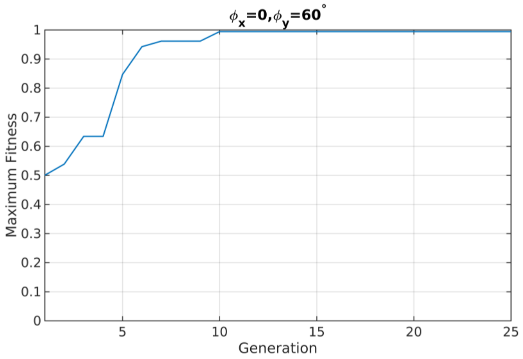
ϕ_x=0,ϕ_y=60°
Maximum Fitness
Generation

(t)

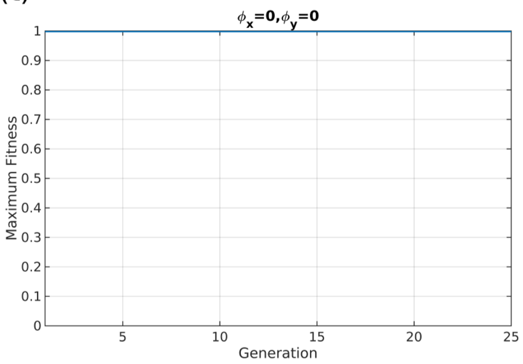
ϕ_x=0,ϕ_y=0
Maximum Fitness
Generation

(u)

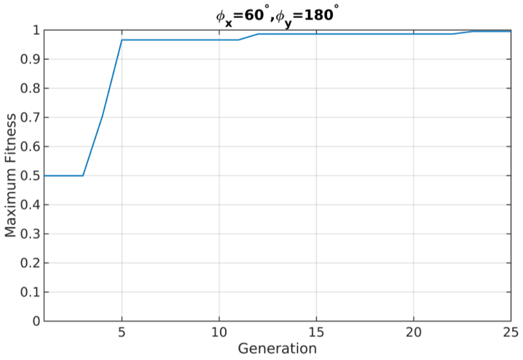
ϕ_x=60°,ϕ_y=180°
Maximum Fitness
Generation

(v)

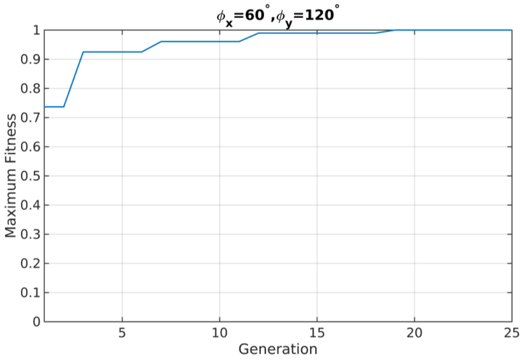
ϕ_x=60°,ϕ_y=120°
Maximum Fitness
Generation

(w)

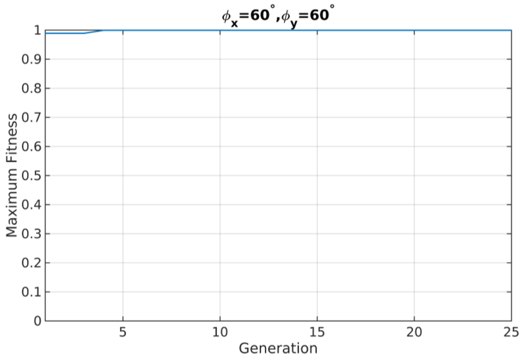
ϕ_x=60°,ϕ_y=60°
Maximum Fitness
Generation

(x)

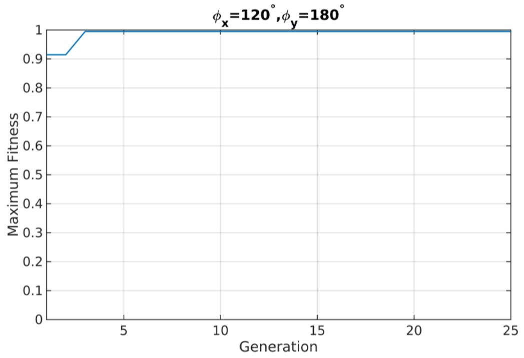
ϕ_x=120°,ϕ_y=180°
Maximum Fitness
Generation

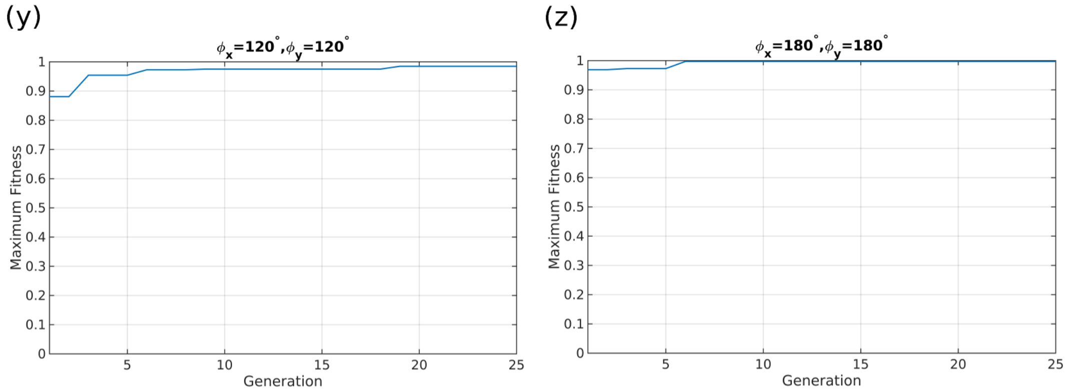

Fig. S-15: (a)-(z) GA convergence plots, corresponding to the compact birefringent all-dielectric metasurface in section 3.3 of the companion paper, showing maximum fitness value of each generation versus the generation index. Fitness values change between 0 and 1.0. Each panel corresponds to one specific retardation phase combination (φ_x,φ_y), for x and y components of the reflected beam, as specified in the title of each panel.

S-5.4 Visible-Transparent Infrared-Emitting/Absorbing Metasurface

S-5.4.1 Optimization Parameters and Statistics

GA parameters and statistics corresponding to the visible-transparent infrared-emitting/absorbing metasurface in section 3.4 of the companion paper are given in table S-7.

Table S-7: GA parameters used for the visible-transparent infrared-emitting/absorbing metasurcace in section 3.4 of the companion paper.

Parameters	Value
N_{par}	52
N_{pop}	52
N_{gen}	30
p_{cross}	0.7
p_{mut}	0.02
T_{ind}	151 s
T_{opt}	65.4 h
GPU Memory	180.2 MB

S-5.4.2 GA Convergence Plot

GA convergence plot corresponding to this application is presented in fig.S-16. As it has been discussed in the companion paper, we have employed a two-stage adaptive optimization procedure for this problem. We just optimize the metasurface based on the IR objective sub-function for the first 15 generations, and then consider a small weight for the visible objective in eq.(8) of the companion paper. The discontinuity in the maximum fitness plot of fig.S-16 at the 15th generation is due to the change in the fitness function based on the adaptive optimization. As it can be seen, an improvement in the overall fitness value in the second phase of the optimization has been achieved. Maximum values of the IR and visible sub-functions in each generation are also plotted

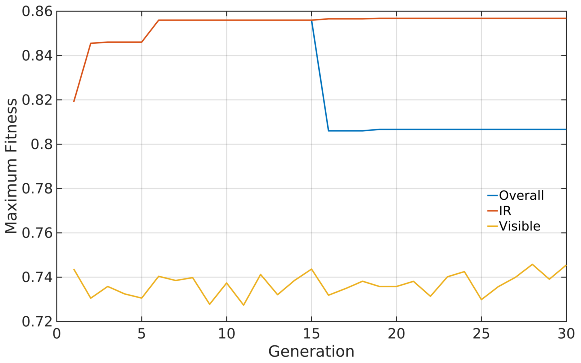

Fig. S-16: GA convergence plot, corresponding to the visible-transparent infrared-emitting/absorbing metasurface in section 3.4 of the companion paper. Fitness values change between 0 and 1.0. The objective function consists of two terms f_{IR} and f_{vis}, corresponding to absorption in the IR and transparency in the visible regimes. Maximum values of f_{IR} and f_{vis} versus generation index are also plotted in the figure. The sudden change in the highest overall fitness value, at the 15th generation, is due to the adaptive optimization.

in the figure. In the first phase of the adaptive optimization, maximum overall fitness is equal to the maximum IR fitness. However, in the second stage, maximum values of f_{IR} and f_{vis} do not necessarily correspond to the maximum overall fitness. As, an individual might possess the maximum f_{IR} value, but not necessarily the highest overall fitness.

S-References

[1] Shaista Babar and JH Weaver. Optical constants of cu, ag, and au revisited. *Applied Optics*, 54(3):477–481, 2015.

[2] A.B. Carlson. *Communication Systems*. McGraw Hill electrical and electronic engineering series. McGraw Hill, 1981.

[3] David L Carroll. Genetic algorithms and optimizing chemical oxygen-iodine lasers. *Developments in theoretical and applied mechanics*, 18(3):411–424, 1996.

[4] Mohsen Farmahini-Farahani and Hossein Mosallaei. Birefringent reflectarray metasurface for beam engineering in infrared. *Optics letters*, 38(4):462–464, 2013.

[5] D.E. Goldberg. *Genetic Algorithms*. Pearson Education, 2006.

[6] Zhengwei Hao. Finite-difference time-domain simulations of metamaterials, January 2013. Accessed : 02-28-2018.

[7] Randy L Haupt. An introduction to genetic algorithms for electromagnetics. *IEEE Antennas and Propagation Magazine*, 37(2):7–15, 1995.

[8] J Michael Johnson and V Rahmat-Samii. Genetic algorithms in engineering electromagnetics. *IEEE Antennas and propagation Magazine*, 39(4):7–21, 1997.

[9] Rei Kitamura, Laurent Pilon, and Miroslaw Jonasz. Optical constants of silica glass from extreme ultraviolet to far infrared at near room temperature. *Applied optics*, 46(33):8118–8133, 2007.

[10] IH Malitson. Interspecimen comparison of the refractive index of fused silica. *Josa*, 55(10):1205–1209, 1965.

[11] Mário G Silveirinha, Andrea Alù, and Nader Engheta. Infrared and optical invisibility cloak with plasmonic implants based on scattering cancellation. *Physical Review B*, 78(7):075107, 2008.

[12] A. Taflove and S.C. Hagness. *Computational Electrodynamics: The Finite-difference Time-domain Method*. Artech House Antennas and Prop. Artech House, 2005.
